# In-depth and high-throughput spatial proteomics for whole-tissue slice profiling by deep learning-facilitated sparse sampling strategy

**DOI:** 10.1038/s41421-024-00764-y

**Published:** 2025-03-11

**Authors:** Ritian Qin, Jiacheng Ma, Fuchu He, Weijie Qin

**Affiliations:** 1 School of Life Sciences, Tsinghua University, Beijing, Beijing, China; 2 State Key Laboratory of Medical Proteomics, Beijing Proteome Research Center, National Center for Protein Sciences (Beijing), Beijing Institute of Lifeomics, Beijing, China

**Keywords:** Proteomic analysis, Proteomics

## Abstract

Mammalian organs and tissues are composed of heterogeneously distributed cells, which interact with each other and the extracellular matrix surrounding them in a spatially defined way. Therefore, spatially resolved gene expression profiling is crucial for determining the function and phenotypes of these cells. While genome mutations and transcriptome alterations act as drivers of diseases, the proteins that they encode regulate essentially all biological functions and constitute the majority of biomarkers and drug targets for disease diagnostics and treatment. However, unlike transcriptomics, which has a recent explosion in high-throughput spatial technologies with deep coverage, spatial proteomics capable of reaching bulk tissue-level coverage is still rare in the field, due to the non-amplifiable nature of proteins and sensitivity limitation of mass spectrometry (MS). More importantly, due to the limited multiplexing capability of the current proteomics methods, whole-tissue slice mapping with high spatial resolution requires a formidable amount of MS matching time. To achieve spatially resolved, deeply covered proteome mapping for centimeter-sized samples, we developed a sparse sampling strategy for spatial proteomics (S4P) using computationally assisted image reconstruction methods, which is potentially capable of reducing the number of samples by tens to thousands of times depending on the spatial resolution. In this way, we generated the largest spatial proteome to date, mapping more than 9000 proteins in the mouse brain, and discovered potential new regional or cell type markers. Considering its advantage in sensitivity and throughput, we expect that the S4P strategy will be applicable to a wide range of tissues in future studies.

## Introduction

Mammalian organs and tissues are highly complex machinery, containing many different microscopic features, and cell types/states. These phenotypically diverse subpopulations contain various kinds of biomolecules organized in spatially defined patterns. The spatial organization of these biomolecules plays a crucial role in determining their function and interaction with their spatial neighborhood in physiological and pathological tissue environments^1,2^. Hence, systematic characterization of the spatial distribution of these molecular features is considered as a prerequisite for deciphering the composition mechanism and intricate network of organs and tissues as well as developing effective diagnostic methods and therapies for disease. Immunohistochemistry (IHC) staining of tissue sections is routinely used in biological research and clinical diagnosis, for visual measurement of tissue morphology, distribution, and expression level of endogenous biomolecules. Although widely used, traditional IHC is only a semi-quantitative method with very limited throughput for measuring multiple targets. Therefore, systemwide, “untargeted” omics methods that enable large-scale spatial measurement of heterogeneous gene expression profiles in various kinds of tissues are highly demanded^1,3,4^. Numerous spatially-resolved genomic and transcriptomic sequencing technologies have been developed that are capable of whole-tissue-slice detection with high spatial resolution and high throughput. Applications of these technologies led to a reconstruction of the genome and transcriptome atlas of various kinds of mammalian organs, elucidation of embryonic development, and a revelation of cellular heterogeneity within spatial context in tumor microenvironment^5,6^. However, it is the gene-encoded proteins that actually regulate essentially all cellular functions and therefore need to be investigated to reveal tissue spatial heterogeneity. Accumulating proteogenomic comparisons revealed that typical Pearson correlation coefficients for mRNA–protein in mammalian tissues are ~0.35–0.6, and can be even lower at single-cell level^7^. Discordant spatial distribution was also revealed by the different zonation patterns of the enterocyte proteins and mRNAs along the villus axis^8^. The poor mRNA–protein correlation may result in biased inference of gene expression distribution using transcriptional data, especially for mammalian brains, considering the large distances between the locations where the proteins are synthesized and transported^9–11^.

To directly measure the spatial distribution of proteome abundance in tissues, a range of mass spectrometry (MS) methods were developed in recent years. Imaging mass cytometry (IMC) using lanthanide metal labeling has single-cell sensitivity and resolution. It has gained significant attention for its high throughput, capable of analyzing thousands of cells per minute. However, similar to IHC, it is also a target-based method that is dominated by antibodies^12^. Only several dozen proteins can be analyzed, which makes IMC less attractive for the discover-driven screening of cellular phenotypes. In contrast, liquid chromatography-coupled biological mass spectrometry (LC-MS), which routinely measures ~10,000 unique proteins from bulk cell and tissue samples, is advantageous in capturing the spatial complexity of tissue proteome. Coupled with visible feature-driven spatial sampling techniques, such as laser capture microdissection, LC-MS-based proteomics enables systemwide study of spatial tissue proteomes with high spatial resolution^1,13–16^. Approximately 2000 proteins can be obtained at 100-µm spatial resolution due to the limited sample amount^17,18^. Although 3000 proteins at 50-µm resolution was reached in a recent work^19^, a larger scale of proteome identification is more desirable to deeply cover regulatory pathways in tissues for spatially resolved physiological and pathological investigation at the molecular level. Although the proteome coverage can be improved using region-specific anatomic dissection of whole tissue, it relies on histologic anatomy structure and cannot provide the high spatial resolution comparable to that of spatial transcriptomics (ST) or metabolomics^20–22^. On the other hand, untargeted spatial strategies^23^ with whole-tissue-slice coverage using “gridding”-like sampling by 3D-printed micro-molds^24^ provide deeper insights into region-specific biomolecule distributions and their correlation with the underlying biological mechanism, without the need for prior knowledge of target proteins, microscopic imaging features, or histologic anatomy structure.

Although more desirable in spatial proteomics studies, whole-tissue slice-based untargeted interrogation is far more challenging, due to the limited sample throughput and sensitivity in current proteomic technologies. First, there is no easy way to uniformly compartment a whole-tissue slice into micrometer-sized samples, while precisely preserving the spatial information. Second, the samples that need to be analyzed expand exponentially with the increased resolution. Taking a circle tissue slice with a diameter of 1 cm as an example, ~8000 samples, corresponding to more than 8000–10,000 h of MS machine time, are required to achieve whole-tissue-slice coverage with a 100 μm resolution, which makes it a formidable task in routine studies. Therefore, whole-tissue-slice-based spatial proteomic approaches that provide reasonable throughput without compromising spatial resolution and proteome depth are urgently needed.

**Fig. 1:**
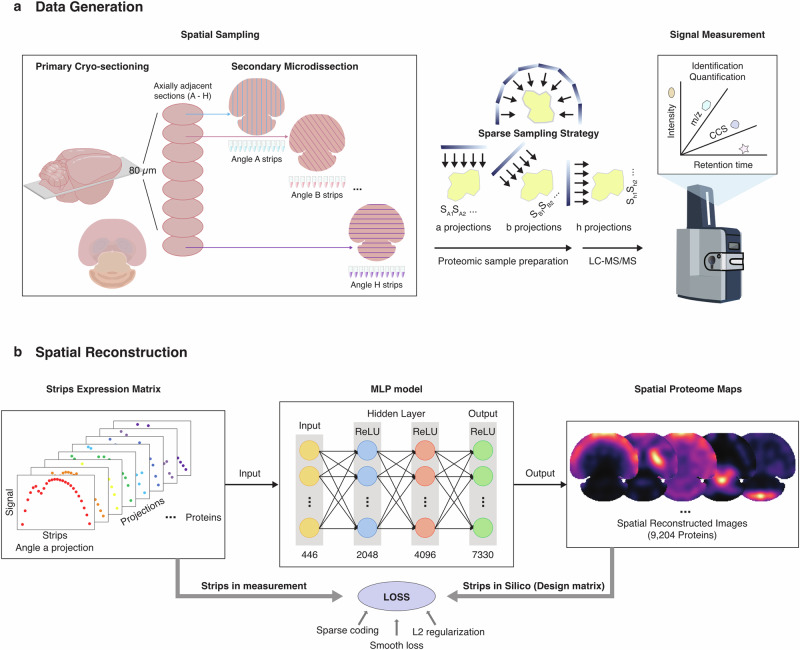
Schematic workflow of the S4P method. **a** S4P sampling and data generation process. **b** Spatial reconstruction processes by DeepS4P.

In this work, we took advantage of the biomedical image reconstruction methods and developed a sparse sampling strategy for spatial proteomics (S4P) that is capable of achieving ~500 μm or higher resolution, while covering centimeter-sized tissue slices within a reasonable MS matching time frame. In S4P, the tissue is cut into consecutive 10-μm thick slices and is further microdissected into a series of parallel strips at pre-defined orientations, resulting in strips spanning the entire tissue slice with different angles for MS-based proteomics analysis. Next, the one-dimensional proteome data from multiple axially adjacent tissue slices are integrated by our sparse sampling strategy to generate sets of signal projections from different angles for image reconstruction to elucidate the spatial distribution of proteins (Fig. 1a; Supplementary Video S1). Image reconstruction methods were previously reported in structural biology and ST^25^ using multi-angle tilt series data for signal superposition to generate three/two-dimensional spatial images of the target molecules. Additionally, medical imaging reconstruction technologies^26–28^, such as magnetic resonance imaging, computed tomography, and positron emission tomography are also widely employed to interpret the raw data (signal) for clinical diagnostics. Numerous algorithms have been developed and applied to image reconstruction, including orthogonal matching pursuit (OMP), compressive sampling matching pursuit (CoSaMP), iterative hard thresholding (IHT), iteratively reweighted least squares (IRLS), generalized basis pursuit (GBP), subspace pursuit, and regularized orthogonal matching pursuit (ROMP)^29–31^.

In this work, we established a multilayer perceptron neural network framework, DeepS4P, which used multi-angle parallel-strip projection data for spatial reconstruction of the MS-detected protein signals to generate two-dimensional proteome abundance and distribution information (Fig. 1b, Materials and Methods). Using S4P, we successfully reconstructed the spatial proteome (SP) of a whole-tissue slice of a mouse brain, consisting of the distribution and expression of 9204 proteins with a spatial resolution of 525 μm (Supplementary Fig. S1). In this way, the MS data acquisition was completed within ~200 h of MS time, half of the time required by the “gridding”-like strategy. More remarkable advantages in sample throughput, detection depth, and method feasibility can be expected using S4P to study samples with larger sizes at higher spatial resolution. For example, an MS time 15–20 times shorter than that of the “gridding”-like strategy can be achieved for a 100 μm spatial resolution (Supplementary Fig. S2). Furthermore, this currently largest SP resource covers major pathways in the mouse brain and includes distribution maps of a substantial number of marker proteins of different anatomical regions and cell types. The first large-scale discovery of different levels of spatial distribution inconsistency between RNAs and proteins in various biological processes, such as synaptic secretion of proteins and neurotransmitters, cellular metabolism, and cognitive & motor function integration, is invaluable for future brain science study.

## Results

### Proteomic sampling, data acquisition, and quality control of the S4P strategy

In S4P, first, eight adjacent 10-µm thick tissue slices were acquired from a mouse brain for primary sectioning. Each tissue slice was further microdissected into parallel strips by Leica laser microdissection (LMD) with their spatial location recorded. A 22.5-degree angle variation in the dissecting direction of the strips was adopted for each slice (Fig. 1a; Supplementary Fig. S3a). The detailed sample spatial assignment of the strips was registered and shown in Supplementary Data S1. Next, each tissue strip was individually collected for LC-MS/MS analysis. Finally, proteome data of the parallel strips in the corresponding locations and striping angles in different slices were projected together to determine the abundance of each protein at a given tissue location using a deep learning model (DeepS4P) for reconstruction (Fig. 1b). S4P takes advantage of a sparse sampling strategy to significantly reduce the number of samples needed to reconstruct the signal, and therefore circumvent the formidable number of samples that need to be analyzed to cover a centimeter-sized whole-tissue slice at high spatial resolution and the throughput limitation of MS-based proteomics. The advantage of using S4P was further elaborated in Supplementary Fig. S2. For ~500 µm spatial resolution achieved in this work, a twice deeper proteome coverage can be achieved while using only half the MS machine time^18^. Even larger advantages up to 15–20 times shorter MS machine time can be expected using S4P for 100 µm spatial resolution while maintaining proteome coverage at ~2000 proteins^17^.

Since hundreds of tissue strips from multiple slices need to be analyzed for proteome spatial mapping, firstly, we evaluated the reproducibility of the Leica LMD-based tissue striping and proteome consistency in the same axial position in adjacent slices. Strips across 90 μm-thick tissue (the 1st, 4th, 7th, and 9th 10 μm-thick slices in a continuous slicing) were dissected in three different areas to ensure sampling diversity (Supplementary Fig. S4a). 8101–8343, 8056–8386, and 7591–8010 proteins were identified in three area groups of strips. Besides the closed scale and highly overlapped protein identification (Supplementary Fig. S4b, c), the box plot of protein quantification in Supplementary Fig. S4d showed uniformly distributed protein abundance in the strips obtained across the 90-μm-thick tissue, indicating the stability of sample striping and proteomic analysis. Quantitative proteomics comparison of the strips in the same axial locations (e.g., S1, S4, S7, and S9 in group A1) showed a high consistency with a low median coefficient of variation (CV) < 0.13 (Supplementary Fig. S4e, f). Principal component analysis (PCA) plot in Supplementary Fig. S4g also revealed highly similar proteomic characteristics among strips from the same axial location, as the strips from the same area group gathered together without overlapping with those from other locations. Further tests on other tissues, such as kidney and liver exhibited a similarly high degree of resemblance within 90 μm along the axial direction (Supplementary Fig. S4h). However, lung tissue displayed a Pearson correlation coefficient variation between 0.75 and 0.95 on the four strips (A1S1–A1S9) in one of the three axial locations, indicating that carefully choosing mapping location may be required. The above results demonstrated high reproducibility of tissue striping and MS analysis and high proteome similarity of the adjacent tissue slices.

Secondly, although our S4P strategy efficiently reduced the number of samples compared to the classic “gridding”-like approaches, ~200 strips (with a strip width of 300 μm) were still required for LC-MS/MS analysis to reconstruct the SP of a mouse brain (Supplementary Fig. S3b). Therefore, robust and reliable data acquisition across all strips via strict quality control is a prerequisite for accurate spatial mapping of tissue proteins. The stability of the LC system was evaluated by spiking synthetic peptides (iRT) that span the entire peptide elution gradient into each tissue strip sample. Variation of retention time was very small across peptide samples of all the tissue strips (Supplementary Fig. S5). Besides the robustness of the LC system, the mass spectrometer that we used was also routinely monitored using tryptic-digested HeLa whole-cell lysate. A minimum of 7000 proteins and 65,000 peptides was required (Supplementary Table S1) to demonstrate good instrument condition. The above results indicated a highly stable performance of the LC-MS/MS system during the whole data acquisition process. In order to minimize batch effects that are commonly observed in large-scale proteome analysis, a sliding window strategy for sample randomization was adopted (Supplementary Fig. S3b). Such a design in the order of sample analysis resulted in a balance between the samples (strips from the same tissue slice) and the groups (different tissue slices)^32,33^. To assess the data quality, we introduced regularly interspersed quality control sample runs to monitor the experimental variability in sample preparation and LC-MS/MS analysis. Highly stable protein identification and quantification (identified 7583–8502 proteins, median CV = 0.16, and Pearson correlation coefficients > 0.93) (Supplementary Fig. S6) of the quality control samples (Supplementary Data S2) were achieved across the whole data acquisition process, again demonstrating the reliability and robustness of the S4P strategy for quantitative proteomic data generation.

**Fig. 2:**
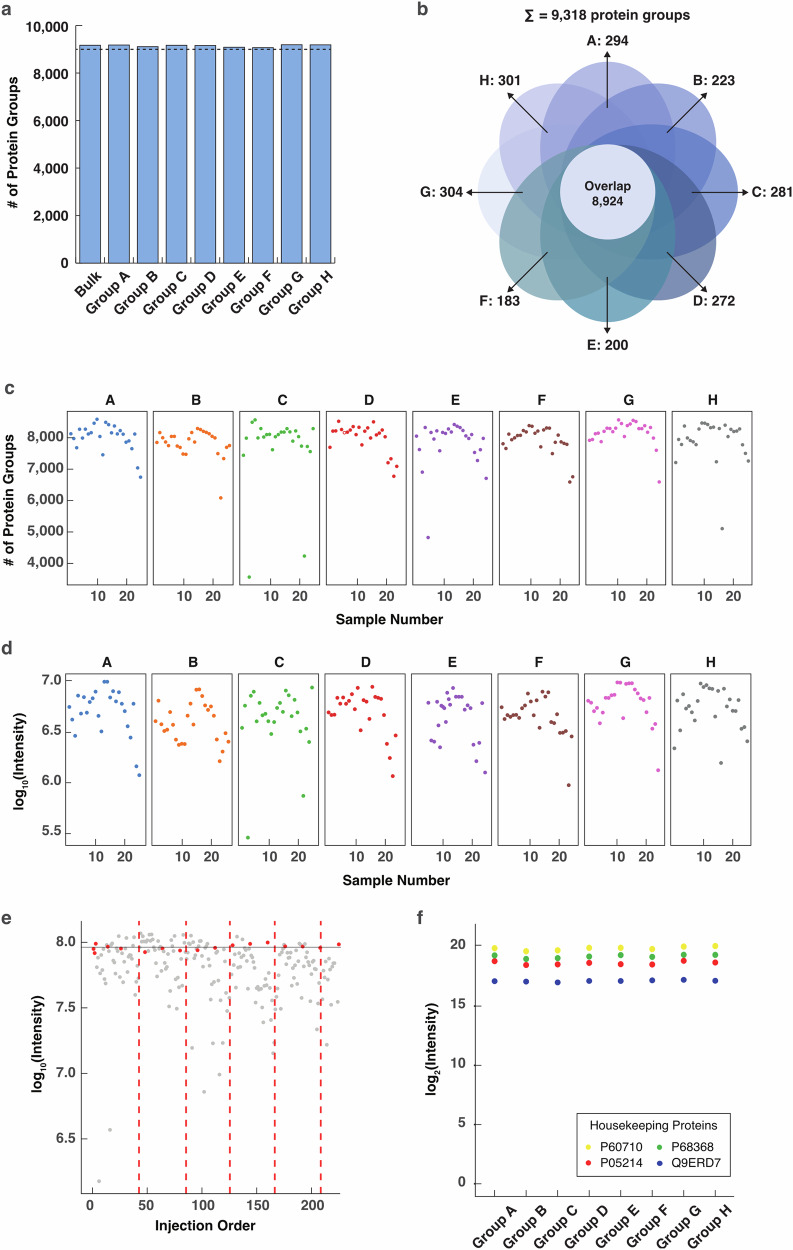
Quality assessment of data generated by the S4P strategy. **a** Bar plot of the identified proteins in each slice. **b** Venn diagram showing overlap of the identified proteins in all the slices. **c** Number of proteins identified in each strip in the corresponding tissue slices. **d** Log_10_-scaled sum of the intensity of all proteins in a strip in the corresponding tissue slices. **e** Intensity distribution of the peptides identified in each strip. The intensity was scaled in log_10_ and the samples were sorted according to the injection order of the LC-MS analysis. The samples are plotted in gray. Quality control samples were displayed in red dots. Different plates (injection vial trays) were separated by red dashed lines. **f** Intensity distribution of the housekeeping proteins. The intensity is log_2_-scaled. The expression levels of the housekeeping proteins in strips are shown in Supplementary Fig. S8.

In this way, a total of 234,768 peptides and 9318 unique proteins (Fig. 2a; Supplementary Data S2) were mapped and quantified in the brain strips. For each slice, the total number of proteins identified was highly closed (9107–9228) and more than 96% of the proteins were shared by different slices (Fig. 2b). Furthermore, ~8000 proteins were obtained from the majority of the strips with only a few outliner strips at the edge of the tissue slices (Fig. 2c), indicating that the major difference in spatial protein distribution in the mouse brain was protein quantity instead of identity. The summed protein abundance displayed a trend similar to that of the protein identification scale across the strips and slices (Fig. 2d). A narrow range between 6.5 and 7 after log_10_ scaling was discovered, with the 1.48% outlines displaying an intensity 0.5–1 order of magnitude lower at the right edge strips (Fig. 2d). In Fig. 2e, the total peptide intensity varied ~0.5 orders of magnitude among strips, while the quality control samples (red dots) were uniformly distributed and highly comparable, again indicating reproducible strip sampling, stable analysis by the LC-MS system and reliable proteome quantification across the strips and slices. The fluctuation among the strips is attributed to their size differences, which can be normalized in subsequent data processing. We further measured the abundance of four endogenous housekeeping proteins, which are supposed to be evenly expressed across the mouse brain. As shown in Fig. 2f and Supplementary Fig. S7, the abundance of the housekeeping proteins was evenly distributed across the slice and in most of the strips. Furthermore, we implemented batch effect evaluation in large-scale proteomic datasets. PCA and UMAP plots showed highly overlapped and randomly distributed proteome quantification without any subgrouping among tissue slices, sample plates, and sample batches (Supplementary Fig. S8), indicating no obvious batch effect in the datasets. The above results further demonstrated the reliability of our experimental design, sampling strategy, and data acquisition for generating high-quality proteomic data.

### SP reconstruction and accuracy evaluation of the mouse brain by S4P

After demonstrating the robustness and reliability of our strategy for generating proteome data with deep coverage and high reproducibility, we developed a deep-learning model (DeepS4P) for spatial reconstruction of the S4P data. In DeepS4P (Fig. 1b), we developed a feedforward neural network for the parallel projections of proteomics data to reconstruct the localization and distribution of proteomes in an unsupervised way. We first used the total intensity of non-endogenous iRT peptides as the spike-in for removing the outlier strips (see Materials and Methods). Then, the protein intensities were normalized by their non-zero median values. A design matrix was constructed, which returns the pseudo strip projections into the expected protein expression. The DeepS4P is trained by the pseudo-expression matrix transformed from a design matrix and the real detected expression data by minimizing the loss function, which consists of sparse coding, smooth loss, and L2 regularization (Fig. 1b, Materials and Methods). In this way, we reconstructed the parallel-slice projection for each protein in the strips and mapped them back to the range of the tissue slices, and obtained 9204 protein distribution maps.

**Fig. 3:**
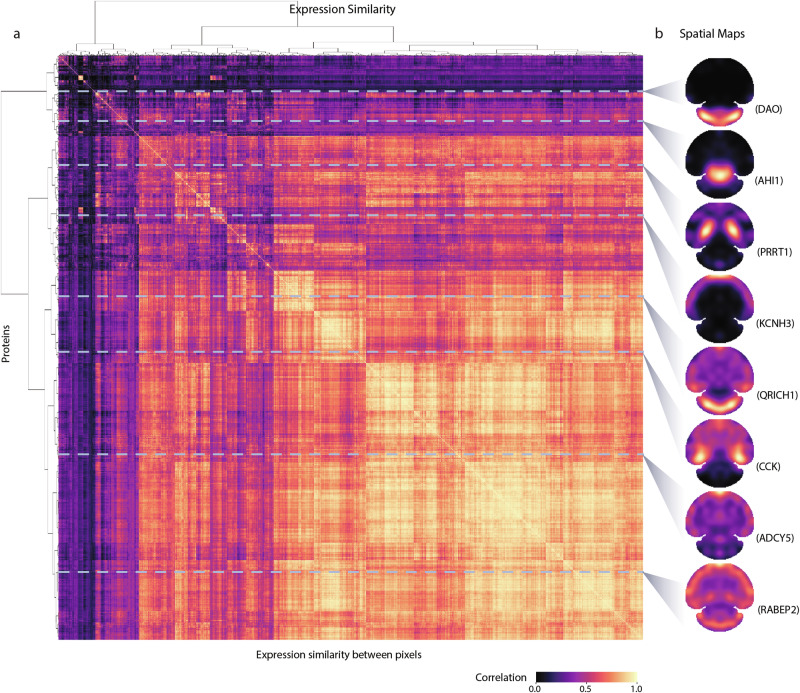
Similarity map of the spatial proteome of the mouse brain. **a** The correlation matrix computed between pixel signals. Ward’s linkage with Euclidean distances was applied to the resulting distance matrix. **b** Visualization of the S4P spatial maps of certain proteins marked by horizontal gray lines.

To investigate the global spatial heterogeneity of these proteins in the mouse brain, we performed a similarity analysis of the S4P data by calculating the pairwise expression correlation using all the pixels in the spatial map of each protein identified by S4P. The pairwise expression correlation is displayed in every row of Fig. 3a by arranging the pixels of each protein simply based on cluster analysis (Ward’s linkage) without dimensional information. In Fig. 3b, the proteins were randomly selected from Fig. 3a, and the pixels were displayed based on their spatial locations. As expected, known proteins located in different anatomical regions, such as DAO in the cerebellum, PRRT1 in the hippocampus, and KCNH3 in the cerebral cortex are well separated in Fig. 3a, indicating that the S4P data can be used to exhibit molecular organization of proteins in omics level. IHC, which is currently considered as the “gold standard” method for in-situ protein expression analysis in a whole-tissue slice (Supplementary Fig. S9), was used to validate the SP distribution reconstructed by S4P. Consistent results between the corresponding SP and IHC images were obtained for proteins displaying different distribution patterns with accurate delineation of anatomical distinctions, indicating no obvious bias of S4P to reconstruct proteins that either concentrate in the center or scatter at the edge of the slices. Furthermore, the entire protein network of highly correlating proteins is provided in Supplementary Data S3. It depicts the correlation of the entire protein maps, which can be used to find proteins showing spatial distribution and expression similar to those of known regional marker proteins of specific regions.

**Fig. 4:**
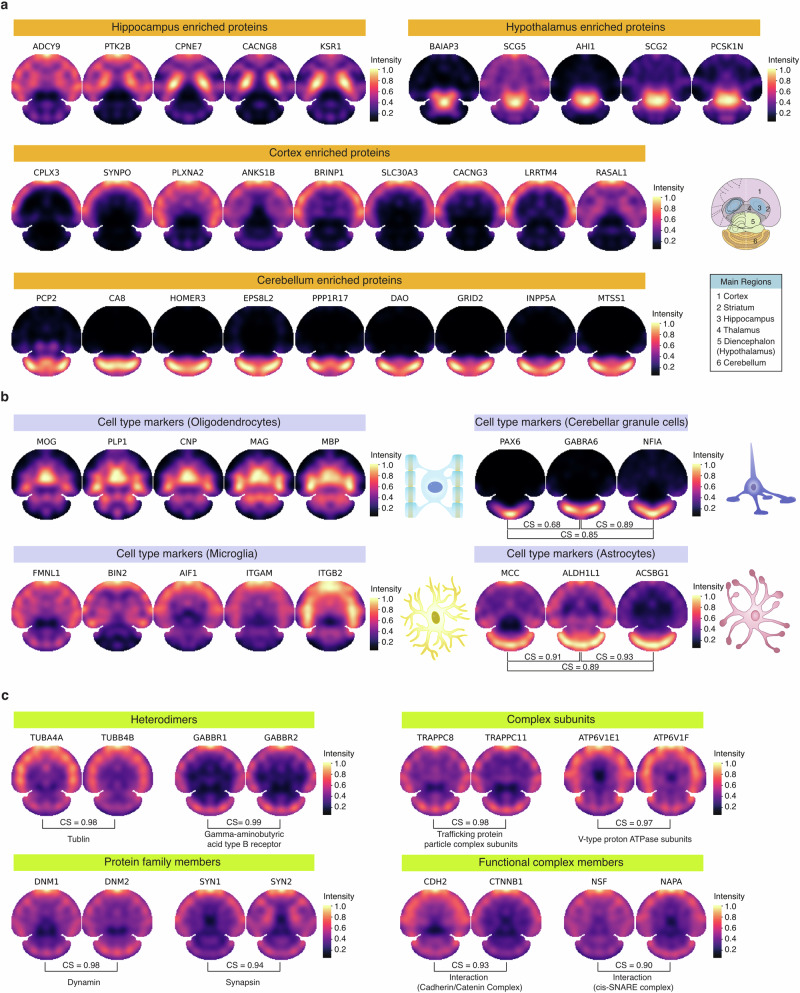
S4P-reconstructed spatial distribution maps of marker proteins and spatially co-localized proteins. **a** Spatial distribution maps of marker proteins of different anatomical regions, including hippocampus, cortex, and hypothalamus. A schematic overview of the anatomical reference annotation for the studied brain section is shown. **b** Spatial distribution maps of known cell-type markers of oligodendrocytes, cerebellar granule neurons, microglia, and astrocytes. **c** Spatial distribution maps of protein pairs that are supposed to be spatially co-localized.

To further evaluate the accuracy of the S4P result, previously reported proteins with distinct region and cell-type enrichment were reconstructed by S4P. Notably, for proteins enriched in the hippocampus, hypothalamus, cortex, and cerebellum, S4P accurately mapped them in their expected regions reported in the literature^21,22,34–37^ (Fig. 4a). For example, using region-resolved mouse brain proteome data^21^, we found that CACNG8 is highly expressed in the hippocampus, which is critical for emotion generation^38^. We then compared the S4P-reconstructed distribution map of CACNG8 with four other hippocampus marker proteins: Copine-7 (CPNE7), Kinase suppressor of Ras 1 (KSR1), Adenylate cyclase type 9 (ADCY9), and non-specific protein-tyrosine kinase (PTK2B) using cosine similarity (CS) for image similarity assessment. The S4P distribution maps of these markers are consistent with that of CACNG8, with high image correlation values of 0.94, 0.96, 0.85, and 0.93 (Supplementary Fig. S10). In addition, we discovered several proteins with distributions that closely align with the known regional markers depicted in Fig. 4a, including NRP1, PRRT1 in the hippocampus, LBHD2, HAP1 in the hypothalamus, KCTD16, SORBS2 in the cortex and GRM1, ARHGEF33 in the cerebellum, which may be potential new brain regional markers. Similar results were obtained for marker proteins of different cell types, such as oligodendrocytes, cerebellar granule neurons, microglia, and astrocytes (Fig. 4b). Highly similar distribution patterns were observed for marker proteins of the same cell type, indicating that S4P correctly reconstructed the spatial location of proteins in various anatomical regions and cell types. We further compared spatial distribution of the cell-type markers reconstructed by S4P with that obtained by an SP study using micro-scaffold-assisted spatial proteomics (MASP) strategy^24^. Despite differences in the tissue slices used and the ways of data acquisition, we found quite similar spatial distribution patterns of the oligodendrocyte markers and cortex markers that were analyzed in both works. The oligodendrocyte markers are all significantly concentrated in the central part of the tissue slice, while the surrounding areas are mostly with very low signal. In contrast, the cortex markers are mainly located at the edge of the slice. The above consistency further demonstrated the reliability of S4P.

We further investigated the S4P-reconstructed maps of known protein groups, which are expected to have identical spatial locations, including heterodimeric proteins, subunits/components of a protein complex, members within a protein family, and proteins that can form a functional complex (Fig. 4c). For example, heterodimeric proteins such as TUBA4A–TUBB4B^39^ and GABBR1–GABBR2^40^ exhibit CS of 0.98 and 0.99, suggesting spatial co-localization. Similarly, as subunits of the trafficking protein particle complex^41^, TRAPPC8 and TRAPPC11 display a high CS of 0.98 for their reconstructed SP images. SP images of ATP6V1E1 and ATP6V1F also show high distribution similarity with a CS of 0.97. These proteins are subunits of the ATP6V1s complex (V-ATPase), which participates in the biological process of transporting hydrogen ions. Both of them are significantly decreased in Alzheimer’s disease, which possibly causes the elevation of lysosomal pH leading to lysosomal disorders^42^. DNM1 and DNM2 from the microtubule-associated force-producing protein family, dynamin, are involved in producing microtubule bundles and binding and hydrolyzing GTP^43^. Their spatial distribution is highly similar with the CS up to 0.98. Targeting Synapsins (Syns) to presynaptic terminals is the key step for signal transmission in the brain. This process involves interactions between different Syn family members like SYN1 and SYN2, which are mainly localized at synaptic sites of mature neurons^44^. In the S4P result, a high spatial distribution similarity between SYN1 and SYN2 is observed (CS = 0.94). Furthermore, we investigated the spatial mappings of protein pairs that form a functional complex, which are also expected to have highly correlated spatial distribution. For example, the E-cadherin/catenin complex plays an important role in epithelial integrity and is involved in intracellular signaling pathways^45^. S4P distribution maps of the two complex members, catenin beta-1 (CTNNB1) and cadherin-2 (CDH2) are highly similar (CS = 0.93). For another example, vesicle-fusing ATPase (NSF) and alpha-soluble NSF attachment protein (NAPA) were reported to form a functional protein complex involved in intracellular vesicle trafficking processes, particularly membrane fusion events^46^. The high localization similarity between them (CS = 0.90) further demonstrated the reliability of S4P for protein spatial distribution mapping.

Although less efficient, a gridding-based spatial proteomics strategy was also used (Supplementary Fig. S2b) to provide “ground truth” of the SP results for further comparison with that obtained by S4P. In the gridding method, the mouse brain tissue slice was cut into small squares with 0.5-mm side length by Leica LMD. Each of the squares was collected with their spatial position recorded and analyzed by LC-MS. This took twice the MS machine time compared to S4P (Supplementary Fig. S2b, c and Data S4). Overall SP comparison between the two methods displayed relatively high consistency with a median CS of 0.869 (Supplementary Fig. S11a). Considering that the two sets of data were obtained from tissue slices of two different mice, some inconsistency is expected. Further comparison of typical proteins obtained by the two methods were shown in Supplementary Fig. S11b. Comparable spatial distribution patterns were observed with CS values no less than 0.8, indicating the reliability of the SP results obtained by S4P. Missing data is another key issue that needs to be addressed in large-scale proteomics studies. No data imputation was used in S4P to avoid possible distortion of the proteome spatial distribution. This highlights the advantage of S4P, in which “large” strips are used for data acquisition instead of “small” squares, therefore, larger sample amounts result in less missing data compared to the gridding method. To further investigate the effect of missing data on the SP reconstruction, we randomly removed the proteome data in 10% and 20% strips. As shown in Supplementary Fig. S12, using protein Cacng8 as an example, no obvious difference was discovered in the protein localization map obtained by DeepS4P after the random strip drop, indicating the capability of S4P for regional pattern extraction to alleviate the missing data-caused deviation in image reconstruction.

### Landscape of protein spatial distribution in the mouse brain

**Fig. 5:**
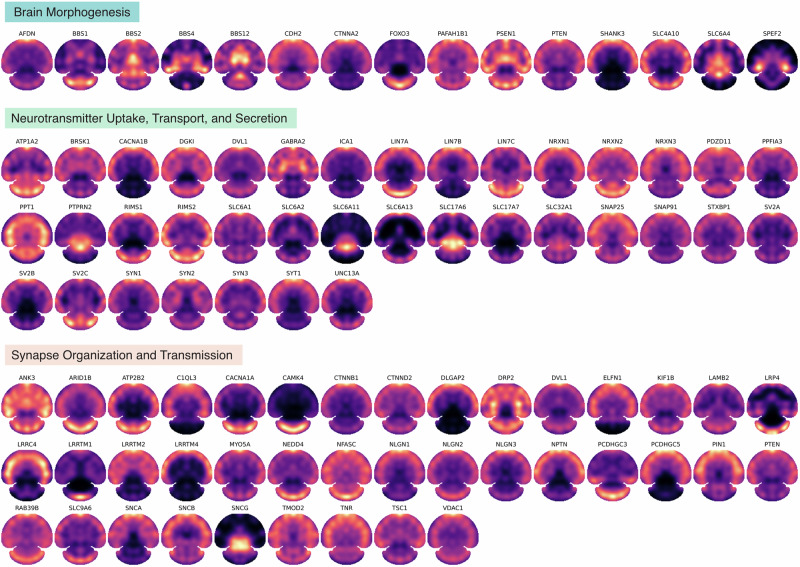
S4P mapping of representative proteins in key biological processes in the mouse brain. The spatial distribution maps of the proteins involved in brain morphogenesis (GO:0048854), neurotransmitter uptake, transport, and secretion (GO:0001504, GO:0006836, and GO:0007269), or neuron–neuron synaptic transmission, regulation of synapse organization, and regulation of synapse organization (GO:0007270, GO:0050807, and GO:0050808).

S4P provides an efficient whole-tissue section proteome mapping technique to achieve the currently deepest SP coverage, with more than 9000 proteins quantified and located at ~500 μm resolution in the mouse brain. The results offer a resource for large-scale exploring protein spatial heterogeneity, which is crucial to extend our understanding of the spatial resolved biological pathway and function regulation in brain science and neurodegenerative diseases. Figure 5 shows the spatial distribution map of typical proteins in the key Gene Ontology (GO) biological processes in the brain, including brain morphogenesis, neurotransmitter uptake, transport, and secretion, neuron–neuron synaptic transmission, regulation of synapse organization, and regulation of synapse organization. For instance, neurotransmitters are chemical messengers that play a fundamental role in communication between neurons in the brain. Neurotransmitters and their uptake, transport, and secretion are crucial for the physiological function of the brain, and dysregulation of neurotransmitters has been discovered in a variety of pathological processes^47^. Spatial proteomic investigation of neurotransmitters has the potential for unraveling the region-specific mechanisms of neurodegenerative diseases, which may enable more precise treatment. Furthermore, the proteins involved in synapse organization and transmission processes were mapped by S4P and shown in Fig. 5 as well. Investigating neuron–neuron synaptic transmission and regulation of synapse organization is crucial for our understanding of how the nervous system functions, how information is processed and transmitted between neurons, and how disruptions in these processes lead to neurological disorders^48^. The regional similarity of these proteins may be attributed to their correlated functional roles that require spatial proximity. For example, a similar distribution pattern with the CS up to 0.99 was found for SV2B and SLC17A7, both of which were reported to play important roles in malignancy, aggressiveness, and recurrence of glioblastoma^49^. Moreover, for synaptic marker genes like *NRXN3* and *SNAP25*^50^, it is not unexpected to find a similar distribution pattern (CS = 0.96), since both of them play important roles in critical synaptic processes for key brain functions. We also observed a certain extent of regional variability among proteins involved in the same biological processes, which is presumably attributed to their potentially different functions in other brain regions.

**Fig. 6:**
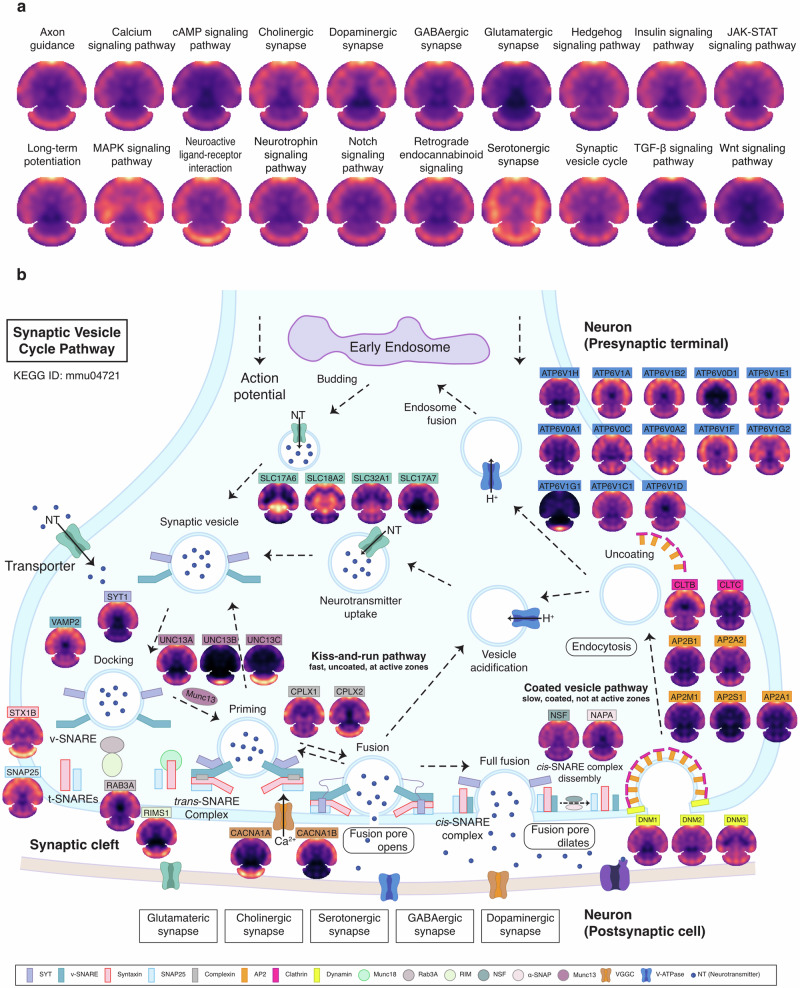
Spatial distributions of proteins in KEGG pathways. **a** Spatial protein expression patterns of key KEGG pathways. **b** Mapping of key proteins involved in the KEGG pathway of synaptic vesicle cycle (ID: mmu04721).

Due to its advantage for whole-tissue slice-based large-scale protein spatial mapping, S4P provides overall perspectives of various signaling pathways, which may potentially facilitate elucidating the spatially regulated brain functions, without the need for prior knowledge of the molecular features or pathology of the tissue. The protein distribution patterns of twenty Kyoto Encyclopedia of Genes and Genomes (KEGG) pathways, which are known to play important roles in brain development, neurotransmission, and signaling processes, were illustrated in Fig. 6a. Using S4P, deep protein component coverage was achieved in multiple pathways, including 66% for SNARE interactions in vesicular transport pathway (KEGG: mmu04130), 59% for GABAergic synapse pathway (KEGG: mmu04727), and 53% for synaptic vesicle cycle pathway (KEGG: mmu04721) (Supplementary Data S5). For example, the synaptic vesicle cycle in Fig. 6b is the key pathway responsible for the transmission of neural signals in the brain. Dysregulation of the synaptic vesicle cycle pathway contributes to synaptic dysfunction and impairments in neurotransmitter release^51^, which are implicated in various brain disorders^48^ and neurodegenerative diseases, such as Alzheimer’s disease, Parkinson’s disease, Huntington’s disease, and autism spectrum disorder^52–54^. Figure 6b shows the distribution maps of representative proteins in the synaptic vesicle cycle pathway acquired by S4P, including V-ATPase transporters, neurotransmitters (SLC17A6, SLC18A2, SLC32A1, SLC17A7), vesicle priming and docking-related proteins (RIMS1, UNC13A, UNC13B, UNC13C), vesicle fusion protein (such as SNARE complex-related proteins (SYT1, VAMP2, STX1B, SNAP25, STXBP1) and complexin proteins (CPLX1, CPLX2)), vesicle recycling and endocytosis proteins (such as dynamin (DNM1, DNM2 DNM3), clathrin (CLTB, CLTC)), vesicle recycling and exocytosis (such as NSF, α-SNAP (NAPA) and Rab GTPases (RAB3A)). Interestingly, diversified spatial distribution patterns were discovered for the above proteins, indicating that the synaptic vesicle cycle pathway is possibly activated in specific brain areas by complex regulating mechanisms. Therefore, future studies using a combination of S4P and time-resolved mouse models may provide information on the dynamic distribution and functional variation of synaptic vesicle proteins under different physiological and pathological conditions for a more comprehensive understanding of synaptic transmission. In this view, S4P presents a novel avenue for providing valuable insights into disease and therapeutic research.

### Correlation analysis of S4P and ST mappings

**Fig. 7:**
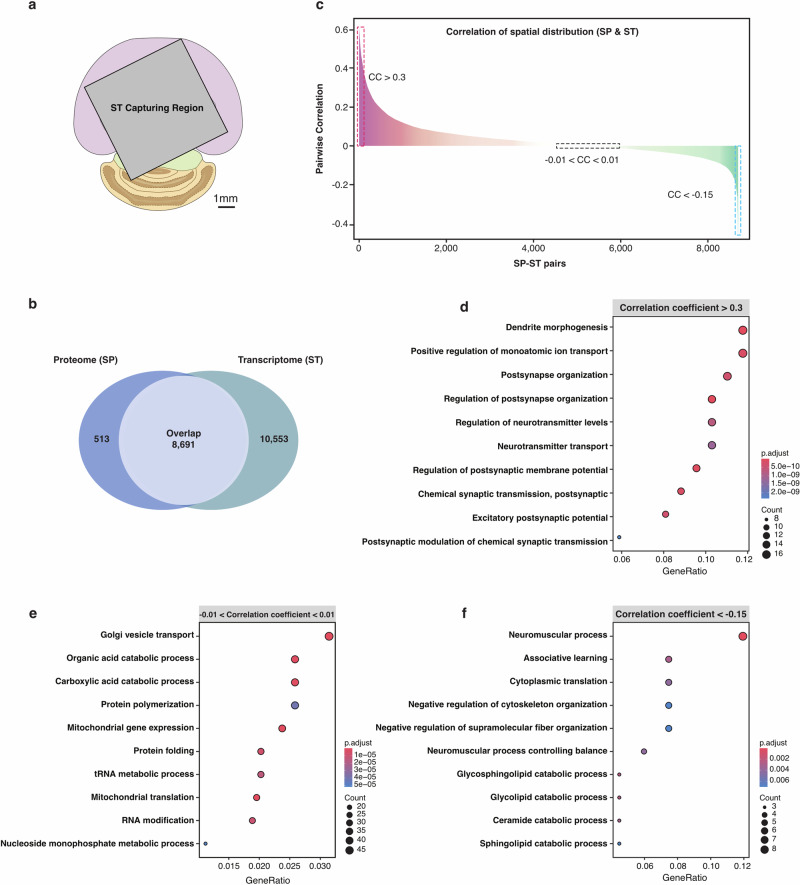
Spatial distribution correlation analysis of transcript–protein pairs. **a** The region captured by ST (mapped in the SP image mask). **b** Venn diagram of SP- and ST-identified gene products. **c** Pearson correlations of the co-detected ST–SP pairs plotted by their correlation coefficient (CC) rank. The red and blue dotted lines indicate the region of CC > 0.3 and CC < –0.15, respectively. The black dotted box represents gene products within the –0.01 < CC < 0.01 range. **d**–**f** The over-representation analysis (ORA) for GO enrichment analysis, showing GO terms for transcript–protein pairs within correlation ranges of (0.3, Max), (–0.01, 0.01), and (Min, –0.15), respectively. Benjamini-Hochberg’s method was used for *P* value adjustment.

We obtained spatial images of 9204 proteins of the mouse brain at the whole-tissue level by the S4P method. Considering that ST is currently the dominant technology for mapping the distribution of gene expression products, we performed ST analysis by Visium from 10X Genomics using a tissue slice from the same mouse brain (Supplementary Fig. S13). Limited by the ST chip size, only a partial region of the tissue slice was used for ST analysis (Fig. 7a). 8691 pairs of gene expression products were found from both ST and spatial proteome SP data (Fig. 7b). We further calculated the Pearson correlation of the transcript–protein pairs for their spatial expression. The correlation between different molecule pairs varies greatly, ranging from –0.47 to 0.62 (Fig. 7c). Typical pairwise SP and ST images of the gene products with different correlation coefficients are shown in Supplementary Fig. S14. Basically, a higher consistency in spatial distribution was obtained for gene products with higher correlation ranking. For gene products with poor correlation, their inconsistency may be attributed to the complex brain tissue, which comprises hundreds to thousands of cell types, each with potentially different spatial distribution patterns of mRNAs and proteins. Next, we performed a classic over-representation analysis for GO enrichment analysis. For the positively correlated transcript–protein pairs (Fig. 7d), the enriched GO terms mainly associate with synaptic regulation and neuronal connectivity, particularly the formation and regulation of synapses, as well as the transport and secretion of neurotransmitters. Co-localization of the synapse-related RNAs and proteins provides further evidence for the so-called local protein synthesis by synaptic transcriptome^55,56^. It has been reported that dendrites and axons of neurons contain a substantial group of localized mRNAs, which encode transmembrane proteins and secretory proteins^57,58^. Local translation is crucial for neurons to overcome the distance constraints and perform rapid regulation of synaptic proteins in responding to outside stimulation. For the spatially non-correlated transcript–protein pairs (Fig. 7e), we found that they were enriched in GO terms related to “Cellular metabolism and protein translation”, including cellular metabolism processes, RNA processing and protein synthesis, and intracellular transport and expression, which underscores the intricate regulation of cellular functions. For the spatially negatively correlated transcript–protein pairs (Fig. 7f), we found that the significantly enriched GO terms were neural regulation and signaling, cellular regulation and catabolism, and cognitive and motor function integration. The spatial discordance between mRNAs and the corresponding functional proteins involved in catabolism processes indicates the necessity of developing SP techniques for exploring the biological mechanism in the brain. Although we cannot fully explain this spatially inconsistent distribution between RNAs and proteins, the results further indicate the advantage of directly mapping protein quantity and location in the tissue.

## Discussion

Although a large number of spatial transcriptomics techniques have been developed and successfully applied in studies on brain function, embryonic development, and tumor microenvironment, direct characterization of the quantity and location of proteins at whole-tissue level is indispensable for both biological function and disease mechanism studies, due to the widely discovered low correlation between RNA and protein. Especially for tissues like the brain, where large-scale proteins are transported away from the sites where they are synthesized, unbiased proteome location measurement is highly demanded to promote elucidation of the spatially organized molecular regulations in the most complex organ. Through the S4P method, we successfully explored the expression and distribution patterns of more than 9000 proteins in the mouse brain without the need for prior structural/imaging knowledge. In addition to the at least twice deeper proteome coverage compared with the previous proteome study using the same spatial resolution^18^, S4P has an obvious advantage over the existing methods in data acquisition time (Supplementary Fig. S2 and Table S2). Via integration of sparse sampling and the newly developed reconstruction algorithm, S4P is capable of large-scale measuring in centimeter-sized samples with the potential to reach micrometer spatial resolution using 5–10 times less MS machine time compared to the current “gridding” methods. Considering that sensitivity and throughput are still the major bottlenecks for SP analysis, greater advantages can be expected using the S4P strategy for larger tissue slices or samples that require higher spatial resolution. Using S4P, we successfully established pixel maps of more than 9000 proteins in the mouse brain, providing a valuable resource for the brain science community. Overall, we anticipate that this largest dataset of spatially resolved mouse brain proteome profiles will be of great use to understand tissue heterogeneity for elucidation of cellular function and disease pathology in the brain.

The current version of S4P is not without limitations. As the first attempt to use a sparse sampling strategy for whole-slice proteomic mapping, we did not choose thin strips, since this would necessitate substantial machine hours for MS analysis. Although LMD is theoretically capable of sample collection at single-cell resolution, the laser damages the adjacent tissue while cutting the target area. For S4P, the damaged area becomes substantial with the reduced strip width. According to our attempts, ~100 µm in strip width is the current limit for LMD-based sample collection. For even higher spatial resolution, a combination of S4P with a tissue expansion method^18^ may be a promising way, in which size of the tissue slice can be evenly expanded ~8 times in every direction to support possible single-cell spatial resolution. Another limitation is that eight adjacent slices are needed for spatial proteome reconstruction currently. Although we did not observe obvious proteome differences among these slices (Supplementary Fig. S4), we still have to consider that the final spatial proteome profile obtained is the average result of the eight slices spanning 80 μm along the *z* axis. Future development of algorithms for sparse sampling-based spatial reconstruction is needed to reduce the slices required, so as to expand the application of S4P to three-dimensional studies.

In this work, we developed the S4P method for spatially resolved and deeply covered proteomic mapping at the whole-tissue slice level. S4P can be used as a complementary strategy to reveal protein distribution that cannot be inferred by spatial transcriptome, especially for the metabolic signaling and protein expression-related genes that are important not only for the understanding of basic biology, but also for the identification of potential targets for future diagnostics and therapies. Considering its advantage in throughput, the S4P method is expected to support spatial proteome for wider applications that allow large-scale investigation of spatial variation of proteins during biological processes, disease progression, and drug treatment for a better understanding of physiological and pathological mechanisms.

## Materials and methods

### Tissue sample preparation

#### Cryo-sectioning

The intact brain of a C57Bl6/J (8-week-old) female mouse was rinsed with PBS on ice and then embedded in optimal cutting temperature compound (OCT), followed by snap-frozen in 2-methylbutane (isopentane) chilled with liquid nitrogen (LN_2_). Next, the mouse brain was cut into a series of 10-μm-thick horizontal sections and deposited on PEN membrane slides (MembraneSlide 1.0 PEN; 415190-9041-000; Carl-Zeiss Microscopy GmBH, Goettingen, Germany). Eight slices were used for strip dissection and proteomic data production, with one for ST data production, and adjacent slices for H&E staining and toluidine blue staining.

#### Laser-microdissection

Tissue strips were microdissected by a Leica LMD7000 system consisting of a microscope with a motorized stage (Leica Microsystems, Illinois, USA). The whole-brain slide was imaged and visualized with the Leica DFC450C microscope camera coupled to a computer. For the S4P sample preparation, adjacent tissue slices were prepared and microdissected into parallel strips with the same strip width. Adjacent tissue sections were microdissected with different angles defined by two feature points in the brain tissue slice, which were aligned across the adjacent tissue slices. In S4P, 300-μm strips (strip widths can be adjusted according to demand) with eight cutting directions were obtained from eight adjacent slices and collected in 200 μL Opaque Adhesive Caps (Carl-Zeiss, Oberkochen, Germany). Each cap was checked under the camera to ensure successful sample collection.

### Proteomic sample preparation for MS analysis

For bulk sample preparation, a whole mouse brain was washed with ice-cold PBS, manually sectioned, and placed into 0.5-mL reinforced polypropylene tubes filled with 1 mm and 3 mm ceramic beads (Cat# 150010 C, Cat# 150030 C). For cell lysis and protein extraction, a mixing lysis buffer (8 M Urea, 1% sodium deoxycholate (SDC), 10 mM Tris-(2-carboxyethyl)-phosphine (TCEP), 40 mM chloroacetamide (CAA), 1× protease inhibitor cocktail (PIC) in 100 mM Tris-HCl, pH 8.5) was added to the tube. The tissue sample was homogenized on the Omni Bead Ruptor Elite (Kennesaw, GA, USA). The lysate was then centrifuged at 12,000× *g* for 20 min at 4 °C, and the supernatant was collected into the Protein LoBind Tubes (Cat# 0030108116, Eppendorf, Hamburg, Germany). The denatured proteins were digested with Lys-C and trypsin in 100 mM ammonium bicarbonate (ABC) at an enzyme-to-substrate ratio of 50:1 (wt/wt) and incubated in a Thermomixer at 37 °C, 1000 rpm for 16 h. The digested peptides were acidified by formic acid (FA) to a pH of 2–3, then subjected to self-made reverse-phase C18 (Empore 3 M) stop-and-go-extraction tips (StageTips)^58^ for desalting and concentration.

For sample preparation of the microdissected strips, 10 μL of lysis (1% SDC, 10 mM TCEP, 40 mM CAA, 1× PIC in 50 mM Tris-HCl, pH 8.5) was added into the cap of collection tubes and invert incubated for 2 h, followed by centrifugation at 5000 rpm for 2 min. The proteins were then digested in 50 mM ABC (pH 8.0) with an enzyme usage of 0.05 pg trypsin per cubic micrometer tissue (pg/µm^3^), and peptides were purified with the Ex kit (OSFP0004-96, Omicsolution, Shanghai, China). All samples were spiked with indexed retention time (iRT) calibration peptides (Ki3002, Biognosys AG, Switzerland) (Supplementary Table S3) to calibrate the retention time of the extracted peptide peaks. Quality control samples were prepared by dissecting mouse brain tissue slices into 20 strips alongside with the actual strips used for spatial proteome reconstruction. The quality control strips were mixed and processed following the same sample treatment procedures as those of the actual strips. The quality control samples were used to evaluate the sample preparation and LC-MS system in every 15–20 sample runs.

### High-pH reversed-phase fractionation for library construction

For spectra library generation by data dependent acquisition (DDA), a whole mouse brain was homogenized, and the proteome was prepared as a mixture, followed by fractionation using high pH separation. Off-line high-pH reversed-phase fractionation was carried out using a RIGOL L-3120 system coupled with an XBridge BEH300 C18 column (150 mm × 4.6 mm i.d., particle size: 5 μm, pore size: 300 Å, Waters) and an XBridge BEH130 PREP C18 guard column (5 μm, 10 mm × 10 mm, Waters). 50 μg of purified peptides were eluted at a flow rate of 0.7 mL/min. The gradient started at 95% solvent A (2% ACN in NH_3_·H_2_O, pH 10) and 5% solvent B (98% ACN with 2% NH_3_·H_2_O, pH 10), and was held for 5 min. Separation was performed over a 64 min elution gradient from 5% to 95% buffer B (0 min 5% B; 5 min 8% B; 40 min 18% B; 62 min 32% B; 64 min 95% B), followed by a 4-min washing step at 5% B and a 8-min washing step at 95% B. The detection wavelength was 214 nm. 60 fractions were collected between 5 min and 64 min, and then pooled into 12 fractions. The 12 fractions were dried in a SpeedVac and resuspended in 0.1% FA; 1 μL of iRT peptides were added before LC/MS analysis.

### LC-MS

To avoid batch effect that commonly exists in large-scale proteomic analysis, we designed to randomize samples in a balanced manner using a sliding window way, which ensured that every slice group (strips direction from “A” to “H”) and the strips in the eight groups (26–28 consecutive strips per group) were analyzed in an orderly interspersed way. In the context of this article, we used the term “Group” to represent a set of strip samples with the same micro-dissected orientation (“A” to “H”), and “Plate” to describe samples in different vial trays. We used the term “Order” when referring to the sample run numbers for MS injection and “Batch” when referring to different batches in the proteomic sample preparation step. Detailed sample information is described in Supplementary Data S1.

The UltiMate 3000 (Thermo Fisher Scientific) liquid chromatography system was connected to the timsTOF Pro2, an ion-mobility spectrometry quadrupole time of flight mass spectrometer (Bruker Daltonics). Samples were reconstituted in 0.1% FA, and 200 ng peptide was separated by analytical column (25 cm × 75 μm i.d., IonOpticks) with a 60-min elution gradient starting at 4% buffer B (80% ACN with 0.1% FA) followed by a stepwise increase to 28% in 40 min, 44% in 5 min, 90% in 3 min and stayed there for 3 min, then decreased to 4% in 3 min and stayed there for 7 min. The column flow rate was maintained at 300 nL/min. For spectra library construction, the instrument was operated in the DDA PASEF mode with ten PASEF scans per topN acquisition cycle and accumulation and ramp times of 100 ms each. MS and MS/MS spectra were recorded from 100 m/z to 1700 m/z, and an ion mobility range (1/K_0_) of 0.6–1.6 Versus/cm^2^ was used. Include charge was set to 0–5; the “target value” was set to 10,000; and dynamic exclusion was activated and set to 0.4 min. The quadrupole isolation width was set to 2 Th for m/z < 700 and 3 Th for m/z > 700. For tissue strip analysis, data independent acquisition (DIA) data was acquired in the diaPASEF mode. We defined 22 × 40 Th precursor isolation windows from m/z 349 to 1229. To adapt the MS1 cycle time, we set the repetitions to variable steps (2–5) in the 13-scan diaPASEF scheme in our experiment. During PASEF MS/MS scanning, the collision energy was ramped linearly as a function of the mobility from 59 eV at 1/K_0_ = 1.6 Vs/cm^2^ to 20 eV at 1/K_0_ = 0.6 Vs/cm^2^.

### Proteomics data processing

Raw Data of DDA and DIA were processed and analyzed by Spectronaut 16.2 (Biognosys AG, Switzerland) with default settings to generate an initial target list. Spectronaut was set up to search the proteome database of Mus_musculus downloaded from Uniprot (UP00000589_10090.fasta, version2022, 21,982 Entries) assuming trypsin as the digestion enzyme. Carbamidomethyl of cysteine was specified as the fixed modification. Oxidation of methionine and N-terminal acetylation were specified as the variable modification. The Q-value (FDR) cutoff on the precursor was applied at 1%, and the Q-value (FDR) cutoff on the protein was 1%. Decoy generation was set to mutated which is similar to scrambled but will only apply a random number of AA position swamps (min = 2, max = length/2). Peptides that passed the 1% Q-value cutoff were used to calculate the major group quantities.

### ST analysis

The slide was stained with filtered 0.1% Mayers Hematoxylin (Sigma, MHS-16) for 10 min and rinsed in cool running ddH_2_O for 5 min. Next, the slide was dipped in 0.5% Eosin (dissolved in 95% Ethanol) 15 times and dipped in ddH_2_O until the eosin stopped streaking. The stained slide was dipped in 50%, 70%, 95%, and 100% ethanol for 1 min each. A 10-μm-thick slide was sectioned. The total RNA was extracted with RNeasy Mini Kit (Part Number: 74104) and the RNA Integrity Number (RIN) was calculated with Agilent RNA 5000 Pico Kit (RIN > 7), assessing the RNA quality of the tissue block. The adjacent slide was then mounted on the Capture Areas of a Visium Gene Expression (GEX) slide.

The fixed tissue section was placed on the slide and the permeabilization enzyme from the Visium Gene expression kit was added. The poly-adenylated mRNA released from the cells was captured by the primers on the spots. Then the slide was placed in the slide cassette following the Visium protocol. 70 μL permeabilization enzymes were added and the slide was placed at 37 °C on the thermocycler adaptor in a thermal cycler with the lid closed. After permeabilization, RT Master Mix was added (reverse transcription reagent) to the permeabilized tissue section to obtain spatially barcoded, full-length cDNA from poly-adenylated mRNA on the slide. Next, the second strand mix was added to the slide to synthesize the second strand cDNA. Subsequently, the cDNA was transferred from each capture area in a new tube. cDNA was then amplified via PCR to generate enough material for library construction. After amplification, cDNA was purified through SPRI-select beads. 1 μL of cDNA was run on an Agilent Bioanalyzer High Sensitivity chip for quality control. Library preparation and sequencing followed the Vision protocol. SpaceRange quality control data was used to correlate tissue RNA integrity status with sequencing data quality (Supplementary Table S4).

### Data pre-processing for reconstruction

After proteomic quantification for each sample (strip), the protein intensities were normalized by the non-zero median values in each sample. Subsequently, we used the total intensity of iRT peptides as spike-in for a heteroscedastic function fit^25^ and to remove the strips that were 1.96 standard deviations below the fit. Next, we used the total intensity of the data for the heteroscedastic function fit and filtered strips that were out of the fit range (Supplementary Fig. S15). To adapt our spatial reconstruction method, we also used the Tomographer used in the STRP-seq analysis to construct a design matrix (*A*)^25^ which contained the spatial information regarding the strip angle and width. The design matrix is a linear operator that acts onto pixel intensities of the reconstructed image to return the expected protein expression into the strip projections. All of the samples from groups A to H were set at the pre-defined angles of –176°, 162°, 139°, 117°, 99°, 72°, 49°, and 27°. In addition, we added two blank strips at both ends of each direction to avoid the influence of the edge effect (sharp signal at the edges of the mask image after reconstruction). To avoid bias during training due to differences in expression levels, each protein was normalized (min–max normalization) and mapped to a range of 0–1.

$${{Pro}}_{{\rm{norm}},i}=\frac{{{Pro}}_{i}-\min ({{Pro}}_{i})}{\max \left({{Pro}}_{i}\right)-\min ({{Pro}}_{i})}$$

### Spatial reconstruction by DeepS4P

All deep learning analysis steps were performed in the Python (version 3.9.13) environment using the Pytorch (version 1.13.1) framework. To learn complex transformation from the proteomic expression matrix of parallel projections to pixel intensities of the reconstructed image, we used a feedforward neural network to model *W*_D_, which consists of three fully connected layers followed by rectified linear unit (ReLU) activations.

$${W}_{{\rm{D}}}=f\left(\theta \right)$$

$$Y={W}_{{\rm{D}}}\left(X\right)$$

Here, *θ* denotes the parameters of *W*_D_. *X* is the proteomic expression matrix of parallel projections. *Y* is the pixel intensities of the reconstructed image. To train our model (*W*_D_) in an unsupervised way, we used the design matrix (*A*) to generate the proteomic expression of pseudostripes (*X*_pseudo_). Subsequently, we could obtain the reconstruction loss (*L*_reconstruct_) by calculating the mean square error of *X* and *X*_pseudo_.

$${X}_{{\rm{pseudo}}}={AY}=A{W}_{{\rm{D}}}(X)$$

$${L}_{{\rm{reconstruct}}}=\frac{1}{m}\mathop{\sum }\limits_{i=1}^{m}{\left({X}_{i}-{X}_{{\rm{pseudo}},i}\right)}^{2}$$

To improve overall stability and robustness of our method, the L2-norm was introduced as weight (*θ*) decay and *λ* was used to control the strength of the regularization effect.

$${L}_{2}=\frac{\lambda }{2}{\Vert \theta \Vert }_{2}^{2}=\frac{\lambda }{2}\mathop{\sum }\limits_{j}{\theta }_{j}^{2}$$

We set two constraints on the pixel intensities: *L*_sparse_ and *L*_smooth_, which were designed to enforce a parsimonious and smooth allocation of the reconstructed signal. *L*_sparse_ is a penalty term based on sparse coding principles to encourage sparsity on the pixel intensities. In addition, *L*_smooth_ is the discrete derivatives of adjacent pixels on the reconstructed image to ensure smoothness.

$${L}_{{\rm{sparse}}}=\alpha {\Vert Y\Vert }_{1}=\alpha \mathop{\sum }\limits_{k}|{Y}_{k}|$$

$${L}_{{\rm{smooth}}}=\beta ({\Vert {D}_{v}Y\Vert }_{1}+{\Vert {D}_{h}Y\Vert }_{1})$$

$$\begin{array}{cc}{D}_{{\rm{v}}}=I\,{\bigotimes}\,{D}& {D}_{{\rm{h}}}=D\,{\bigotimes}\, I\end{array}\,$$

Specifically, *D* is defined as the 1–d discrete derivative operator. *D*_v_ and *D*_h_ are the linear operators to calculate respectively the vertical and horizontal discrete derivatives of the reconstructed image. *α* and *β* determined the sparsity and smoothness of the reconstructed image. The final loss is defined as ${L}_{{\rm{total}}}\,=\,{L}_{{\rm{reconstruct}}}\,+\,{L}_{2}\,+\,{L}_{{\rm{sparse}}}\,+\,{L}_{{\rm{smooth}}}$ and *θ* can be estimated by minimization of *L*_total_. For the final images presented, the scipy.ndimage.filters.gaussian_filter function was applied to apply Gaussian blur for image smoothing.

## Supplementary information

Supplementary Information

Supplementary Data S1_Sample Information

Supplementary Data S2_Strips Identification

Supplementary Data S3_The correlation matrix of entire protein maps

Supplementary Data S4_Gridding Data

Supplementary Video S1.mp4

Supplementary Data S5_KEGG

## Data Availability

All data presented in this study are available in Supplementary information. The LC-MS raw data used for the mouse brain SP reconstruction have been deposited in the iProx with the dataset identifier: IPX0007383000. The raw data of spatial transcriptomic sequencing generated in this study have been deposited in the NCBI Sequence Read Archive (SRA) under the BioProject of PRJNA1033786.

## Code availability

Details of the spatial proteomics analysis, reconstruction, and correlation analysis between ST and SP pipelines can be found in our GitHub repository (https://github.com/Ma-JC/DeepS4P/tree/main).
