# Supplementary material for: In-depth and high-throughput spatial proteomics for whole-tissue slice profiling by deep learning-facilitated sparse sampling strategy
Source: Cell Discov. 2025 Mar 11;11:21. doi: 10.1038/s41421-024-00764-y (PMC11894098; doi:10.1038/s41421-024-00764-y)
Supplement: Supplementary file 1 — Supplementary Information [file 41421_2024_764_MOESM1_ESM.pdf]

Supplementary Information

In-depth and High-throughput Spatial Proteomics for Whole Tissue Slice Profiling by Deep-learning Facilitated Sparse Sampling Strategy (S4P)

Ritian Qin[1,2#], Jiacheng Ma[1,2#], Fuchu He[1,2]* and Weijie Qin[2]*

1. School of Life Sciences, Tsinghua University, Beijing, 100084, Beijing, China

2. State Key Laboratory of Medical Proteomics, Beijing Proteome Research Center, National Center for Protein Sciences (Beijing), Beijing Institute of Lifeomics, Beijing 102206, China

# These authors contributed equally to this work.

* aunp_dna@126.com and hefc@bmi.ac.cn

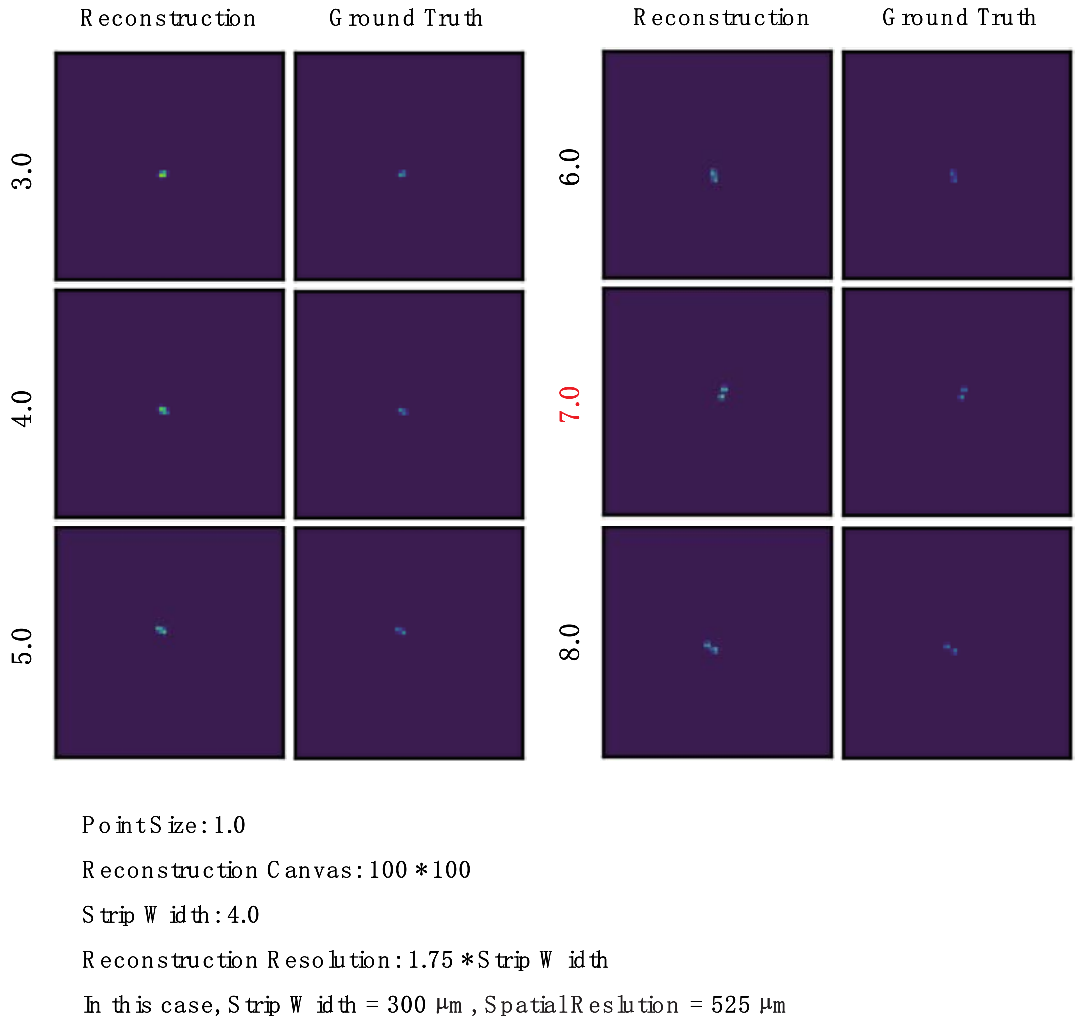

Fig. S1 Spatial resolution calculation for the S4P method.
Assessment of two-point discriminative power and resolution. The two-point effective resolution is defined as the discriminative distance for the reconstructed images. The point sizes were set as 1 and plotted on the square canvas with 100 sides. In our data, the strip width was (100 / 26 samples) approx.4. Under this setting, two points can be separated with a distance of 7, which is 1.75 times the strip width (4). Resolution is determined to be 1.75 times the strip width. As the strip width is 300 μm in this study, the calculated spatial resolution is 525 μm.

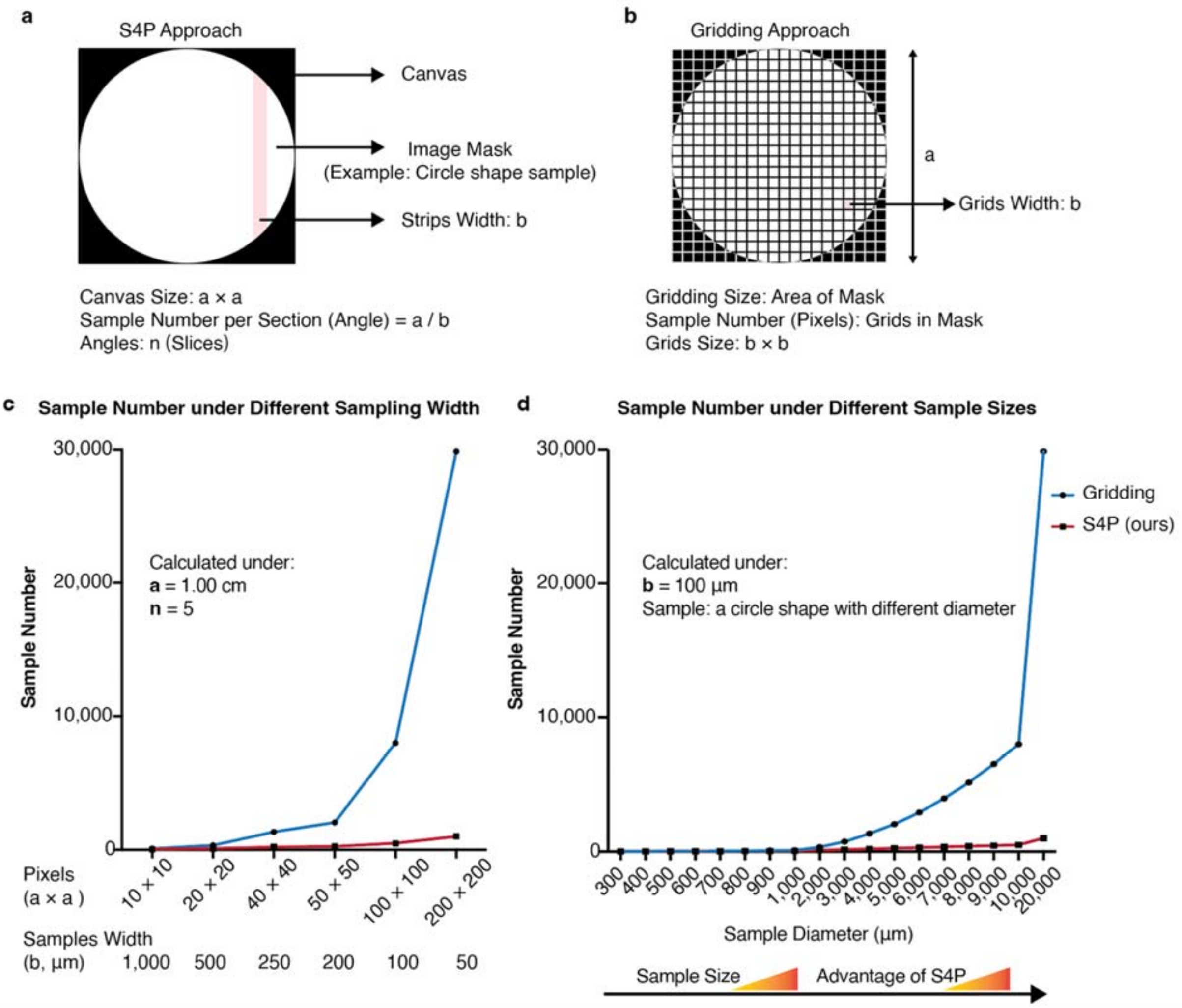

Fig. S2 Comparison of S4P to the existing gridding approach in sample throughput.
a Schematic diagram and parameters involved in the S4P method.
b Schematic diagram and parameters involved in the gridding-like methods.
c Sample number calculation under different sampling widths.
d Sample number calculation for different sizes of tissues slice with a sampling width of 100 µm.

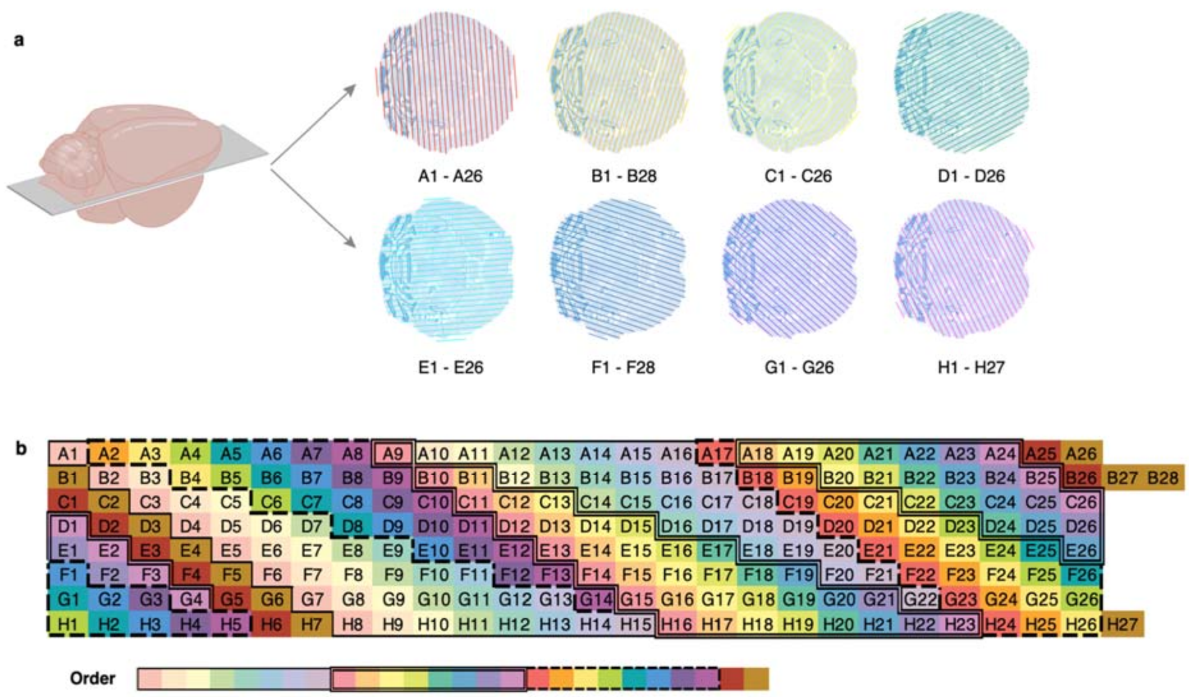

Fig. S3 Experimental design for minimizing batch effects.
a Striping angles in different tissue slices.
b Sliding windows of sample injection order for mass spectrometry analysis.

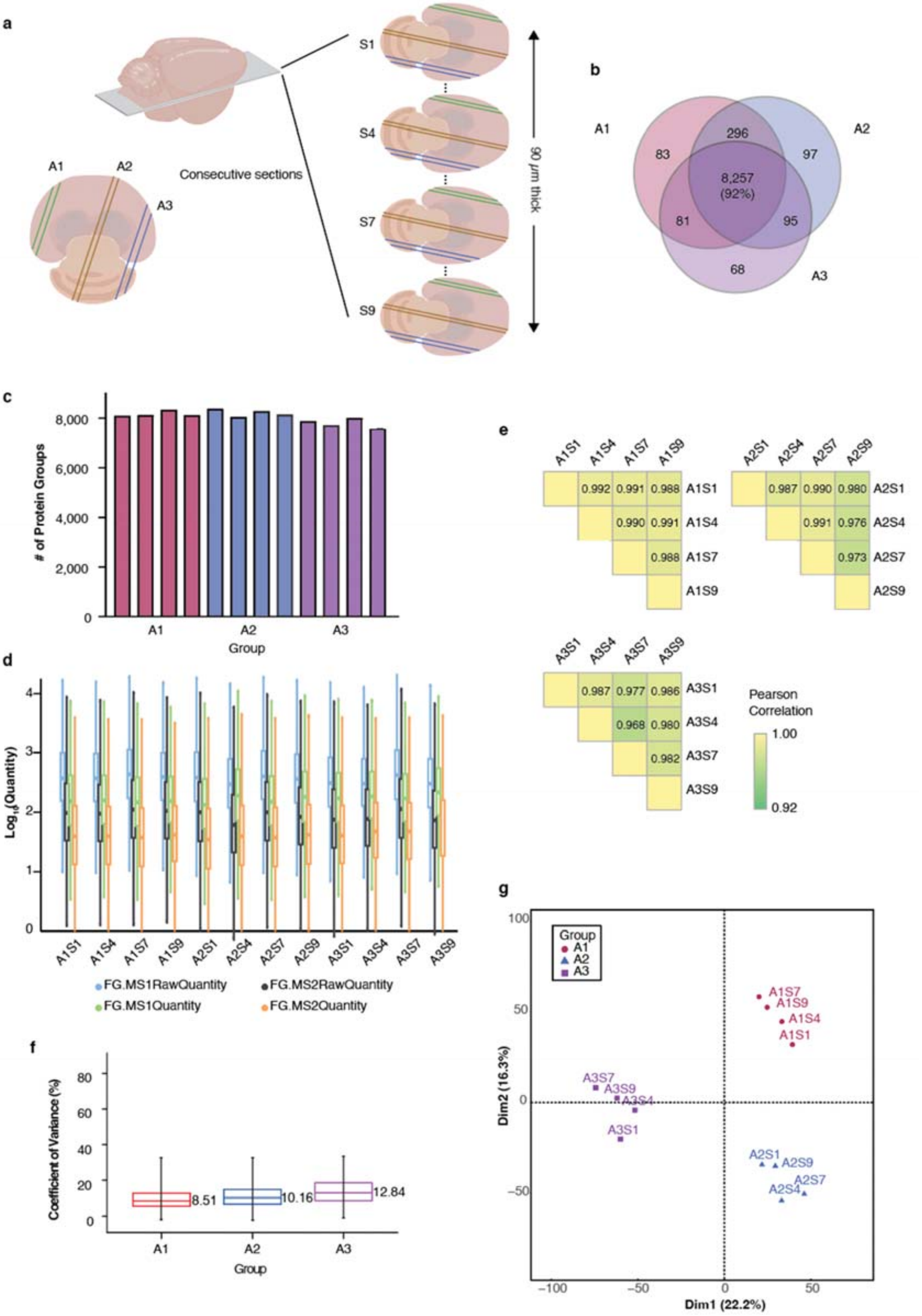
a
S1
S4
S7
S9
90 μm thick
A1
A2
A3
Consecutive sections
b
A1
A2
A3
83
296
97
8,257
(92%)
81
95
68
c
# of Protein Groups
8,000
6,000
4,000
2,000
0
A1
A2
A3
Group
d
Log10(Quantity)
4
3
2
1
0
A1S1
A1S4
A1S7
A1S9
A2S1
A2S4
A2S7
A2S9
A3S1
A3S4
A3S7
A3S9
FG.MS1RawQuantity
FG.MS2RawQuantity
FG.MS1Quantity
FG.MS2Quantity
e
A1S1 A1S4 A1S7 A1S9
0.992 0.991 0.988 A1S1
0.990 0.991 A1S4
0.988 A1S7
A1S9
A2S1 A2S4 A2S7 A2S9
0.987 0.990 0.980 A2S1
0.991 0.976 A2S4
0.973 A2S7
A2S9
A3S1 A3S4 A3S7 A3S9
0.987 0.977 0.986 A3S1
0.968 0.980 A3S4
0.982 A3S7
A3S9
Pearson Correlation
1.00
0.92
f
Coefficient of Variance (%)
80
60
40
20
0
8.51
10.16
12.84
A1
A2
A3
Group
g
Group
A1
A2
A3
A1S7
A1S9
A1S4
A1S1
A3S7
A3S9
A3S4
A3S1
A2S1
A2S9
A2S7
A2S4
100
50
0
−50
Dim2 (16.3%)
−100
−50
0
50
Dim1 (22.2%)

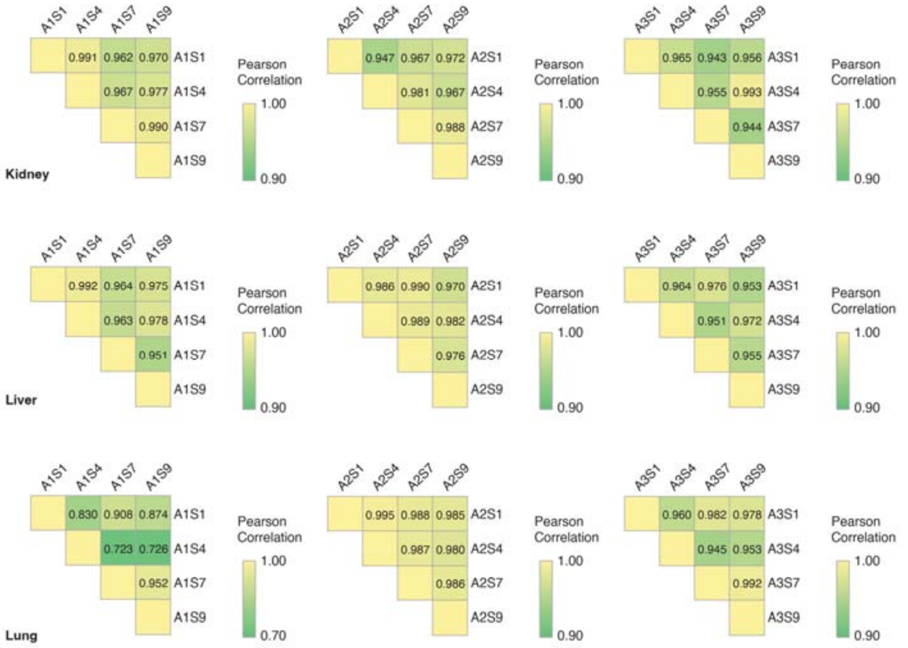

Fig. S4 Proteome consistency in the same axial position in adjacent slices across 90-µm-thick tissue

a Schematic overview of the test samples microdissected in 90 µm thick tissue. A1, A2, and A3 represent three different areas in the center, left and right side of the slices. From nine consecutive 10-µm-thick sections, S1, S4, S7, and S9 were collected for test.

b Venn diagram of protein identification in the A1, A2 and A3 groups.

c Bar plot of the identified proteins in each sample.

d Box plot of raw proteomic data quantity distribution.

e Pearson correlation of the brain samples.

f Coefficient of variance (CV) distribution of the A1, A2 and A3 groups, and median CV of different groups are labeled.

g Principal component analysis (PCA) of the test samples.

h Pearson correlation of the kidney, liver and lung samples

a

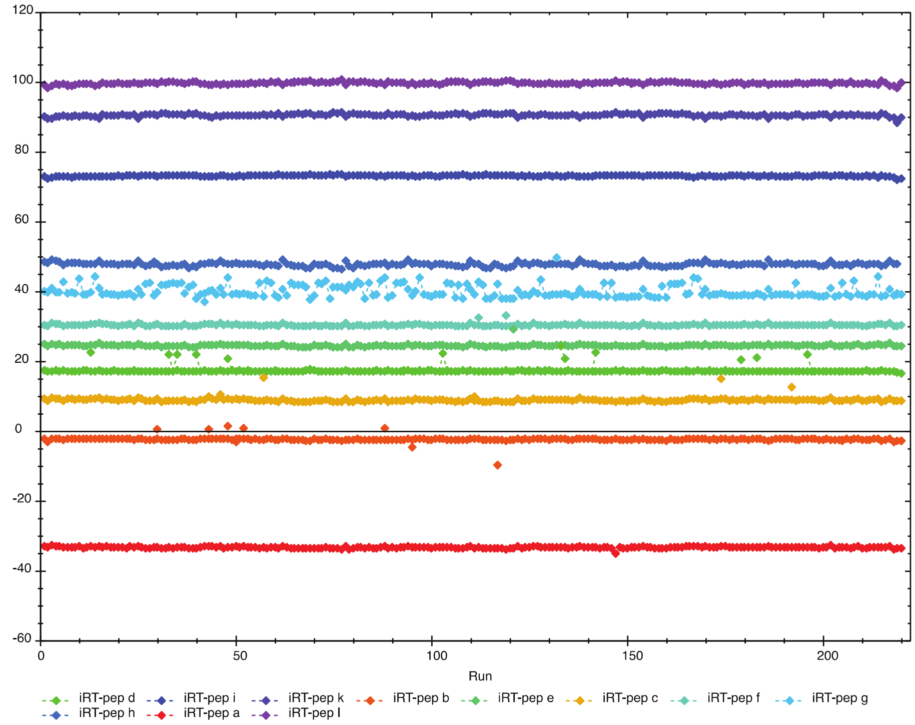

b

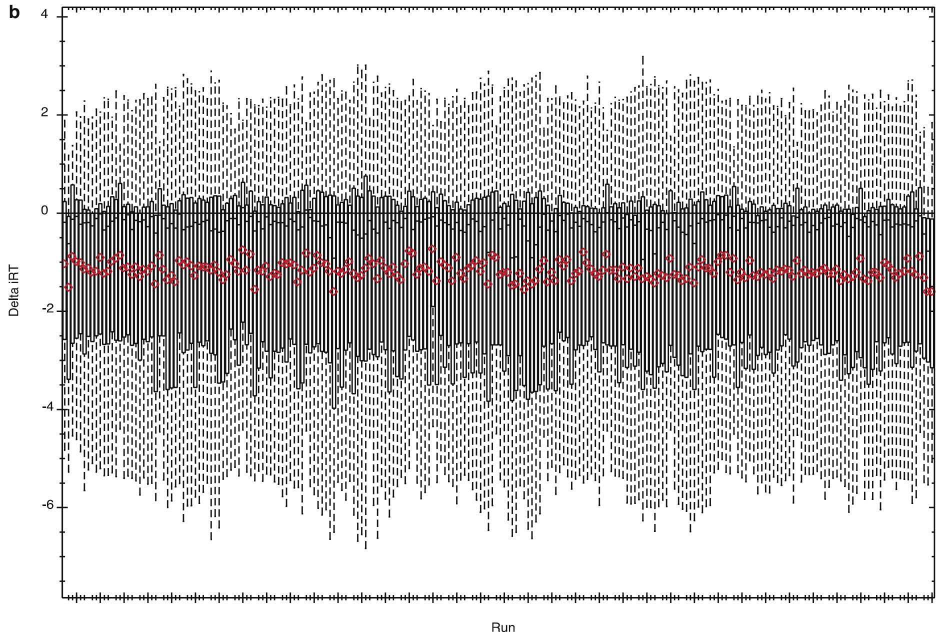

Fig. S5 Retention time distribution of the iRT peptides in all the MS raw data
a Retention time of the non-endogenous iRT peptides (listed in Table S2)
b Delta retention time of the iRT peptides showed low variation across all samples.

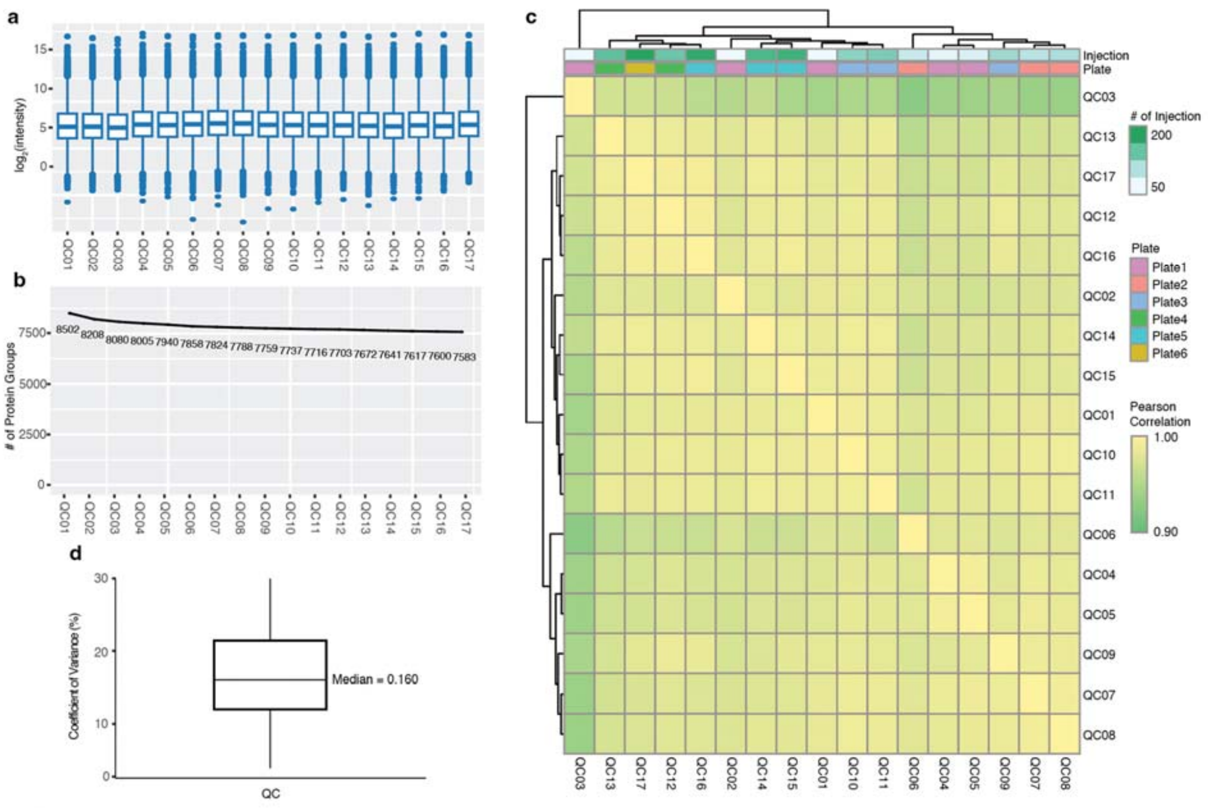

Fig. S6 QC samples for instrument performance monitoring.

a log10 scaled protein intensity of the QC samples.

b Identified proteins in the QC samples, showing stability of the MS condition during the data acquisition process.

c Pearson correlation of the quantified proteins in the QC samples. All the QC samples showed high correlations (r > 0.93).

d Coefficient of variance (CV) distribution of the QC samples, with a median CV of 0.160.

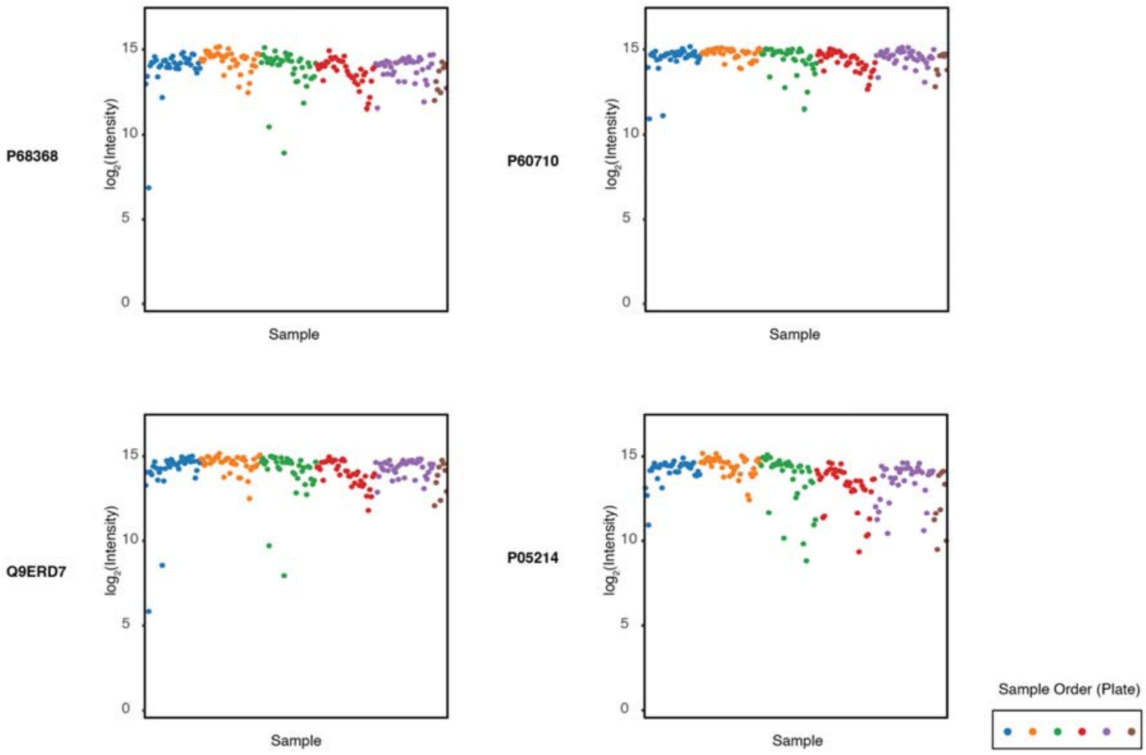

Fig. S7 Quantification of the housekeeping proteins by MS
UniProt entry ID: P68368, P60710, Q9ERD7, and P05214.

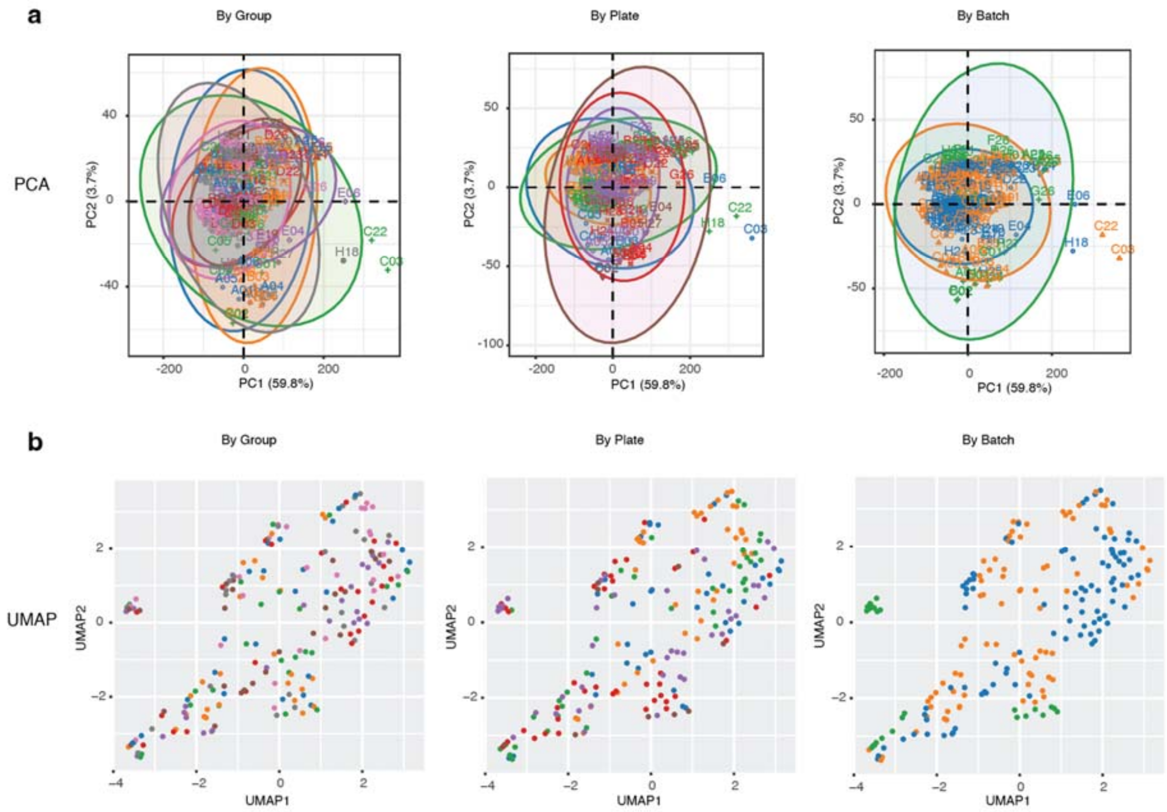

Fig. S8 Batch effect evaluation of the proteomic data

a PCA and **b** UMAP plots of proteomic quantification of the samples by tissue slices, sample plates, and sample batches.

“Group” is a set of strip samples with the same micro-dissected orientation in one tissue slice (“A” to “H”); “Plate” is the sample in different vial trays (MS injection plates); “Batch” is batches in the proteomic sample preparation step.

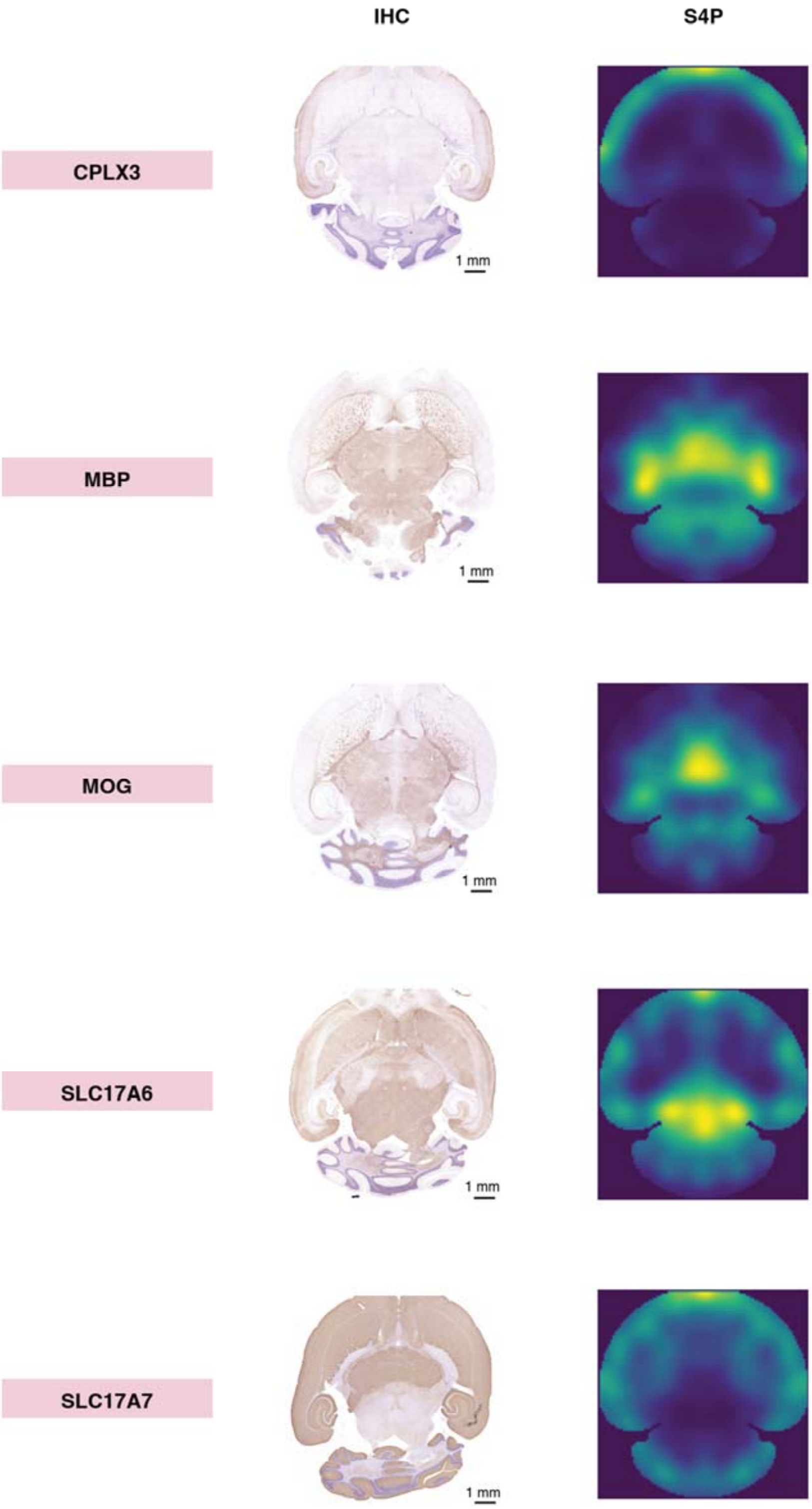

Fig. S9 S4P reconstruction of protein expression patterns and validation by IHC.

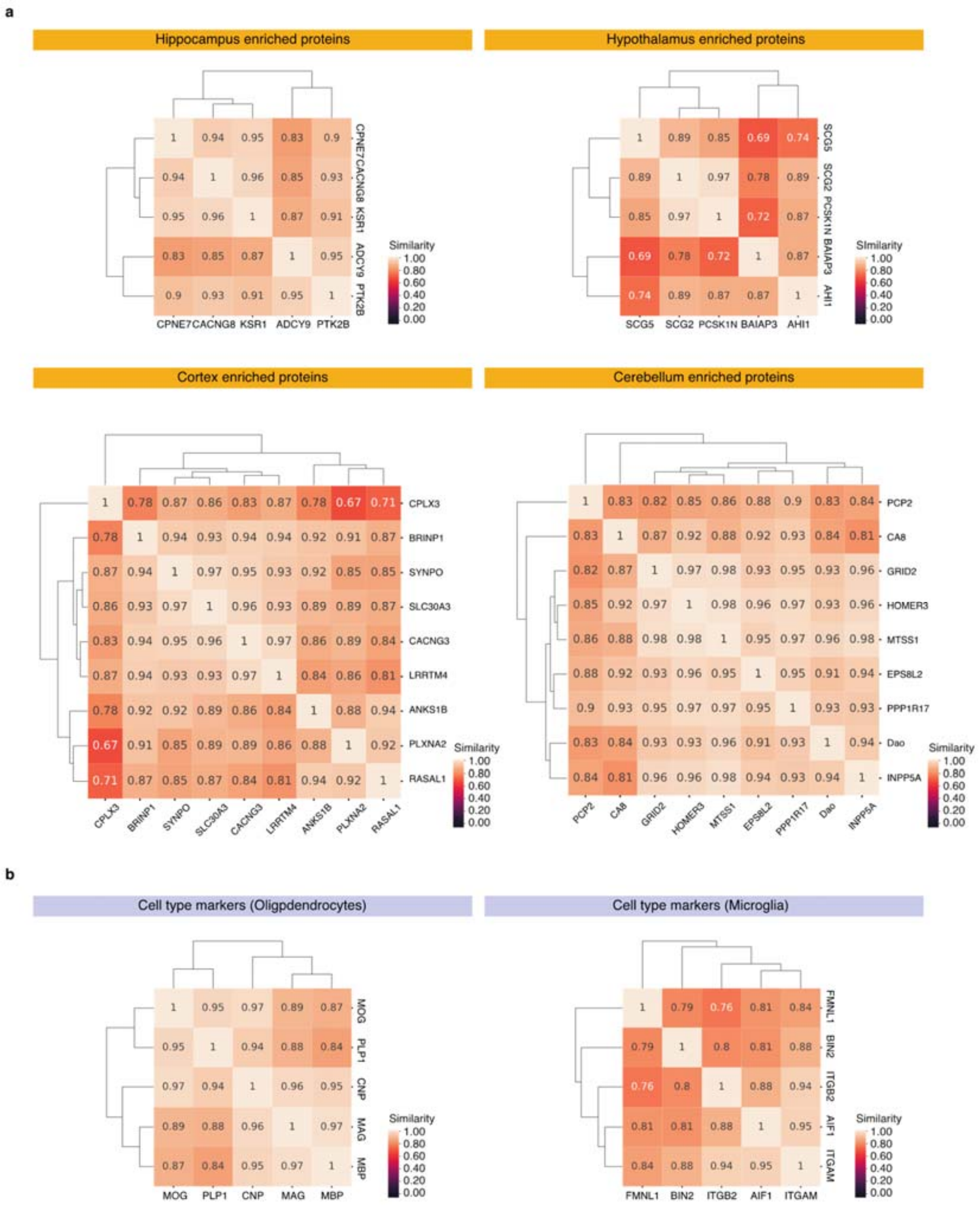

Fig. S10 Cosine similarity of the spatial distribution maps of **a** the mouse brain regional marker proteins and **b** the cell-type marker proteins.

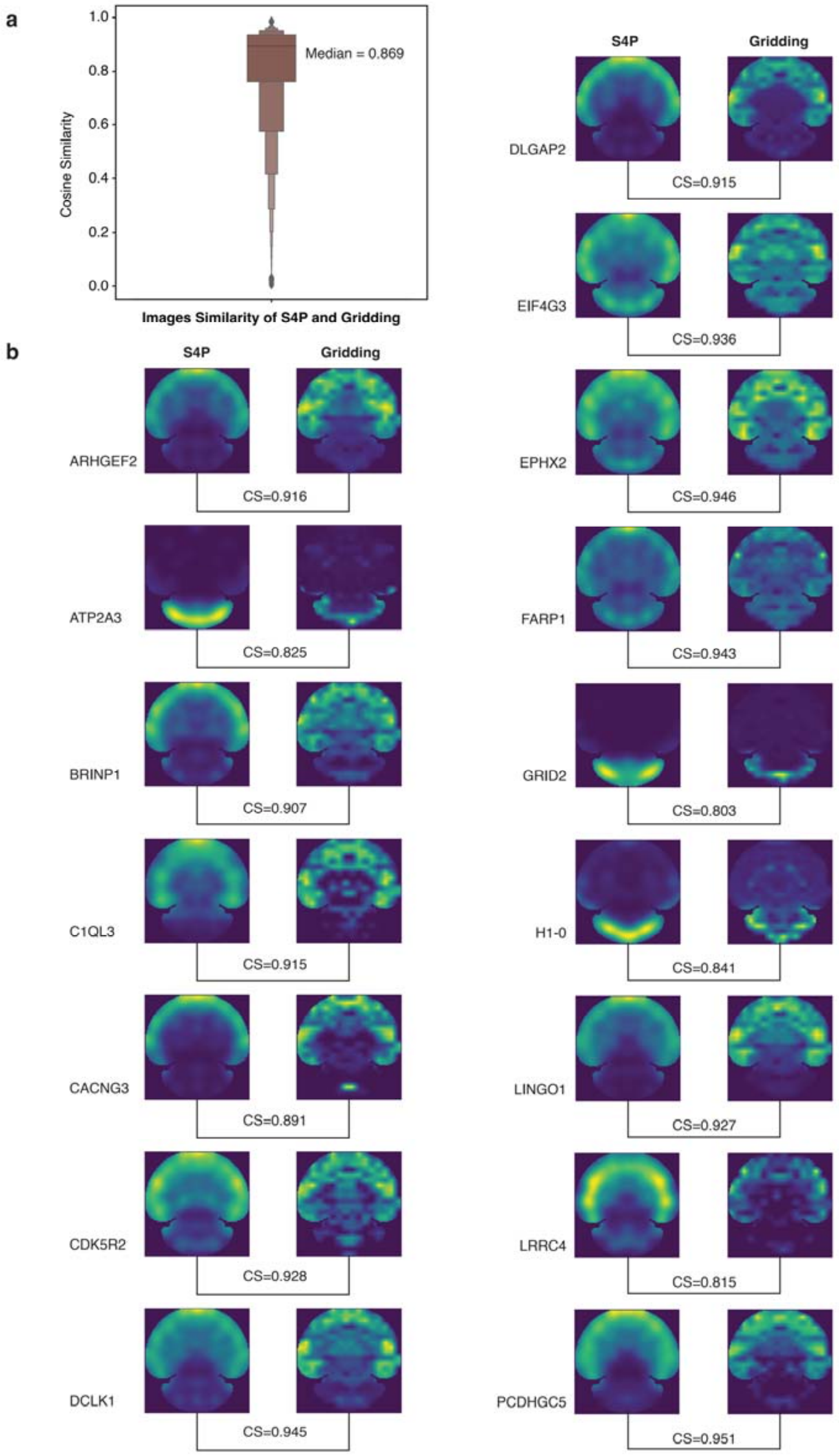

Fig. S11 (a) Global cosine similarity between spatial images obtained by S4P and the gridding method. (b) Spatial distribution images of typical proteins reconstructed by S4P and the gridding method.

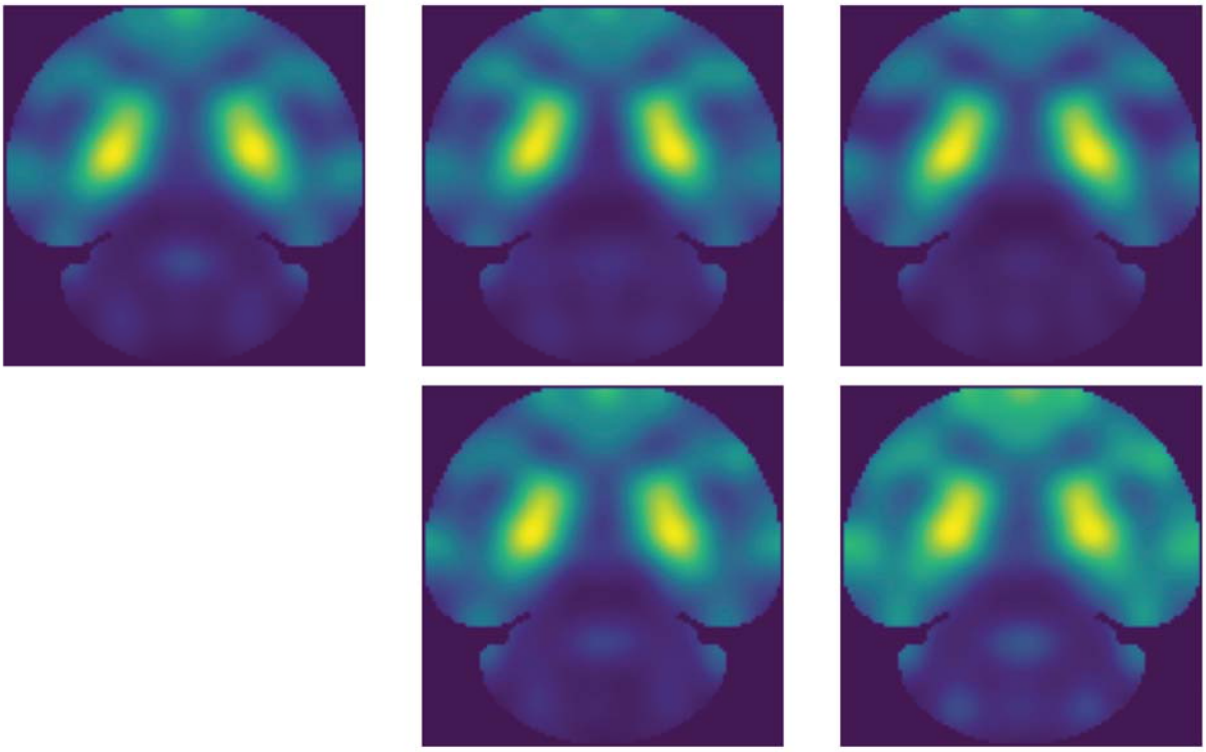

Fig. S12 Evaluation of missing values in the protein localization map reconstruction (Example: Cacng8). In the upper row: S4P reconstructed Cacng8 using 0%, 10% and 20% random strips drop. In the lower row, Cacng8 with different 10% and 20% random strips drop.

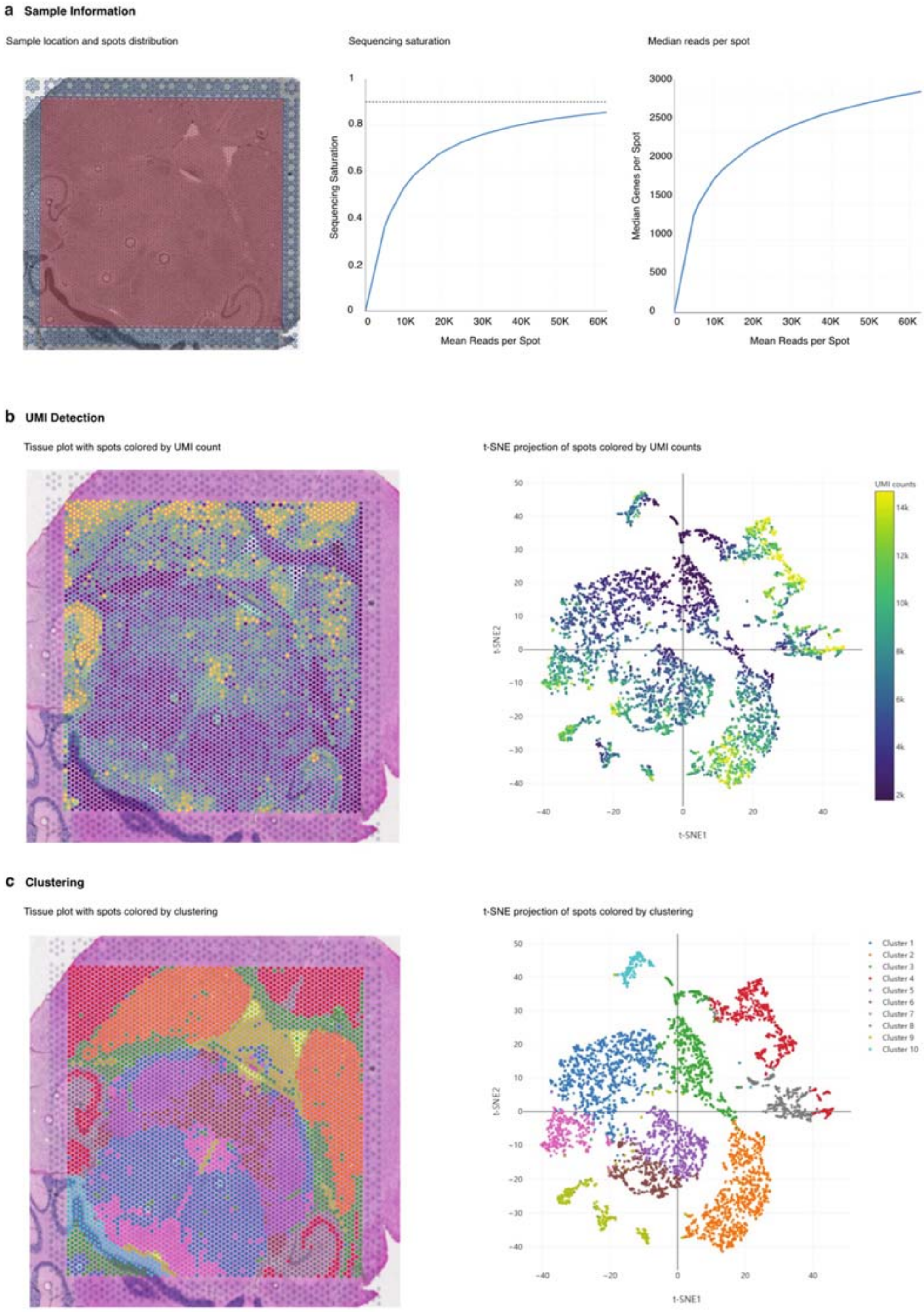

Fig. S13 Spatial transcriptomics data generation.
a ST Sample information of the detected regions and transcript reads per spot.

Tissue plots t-SNE projection by **b** UMI count and by **c** clustering with 10X standard workflow.

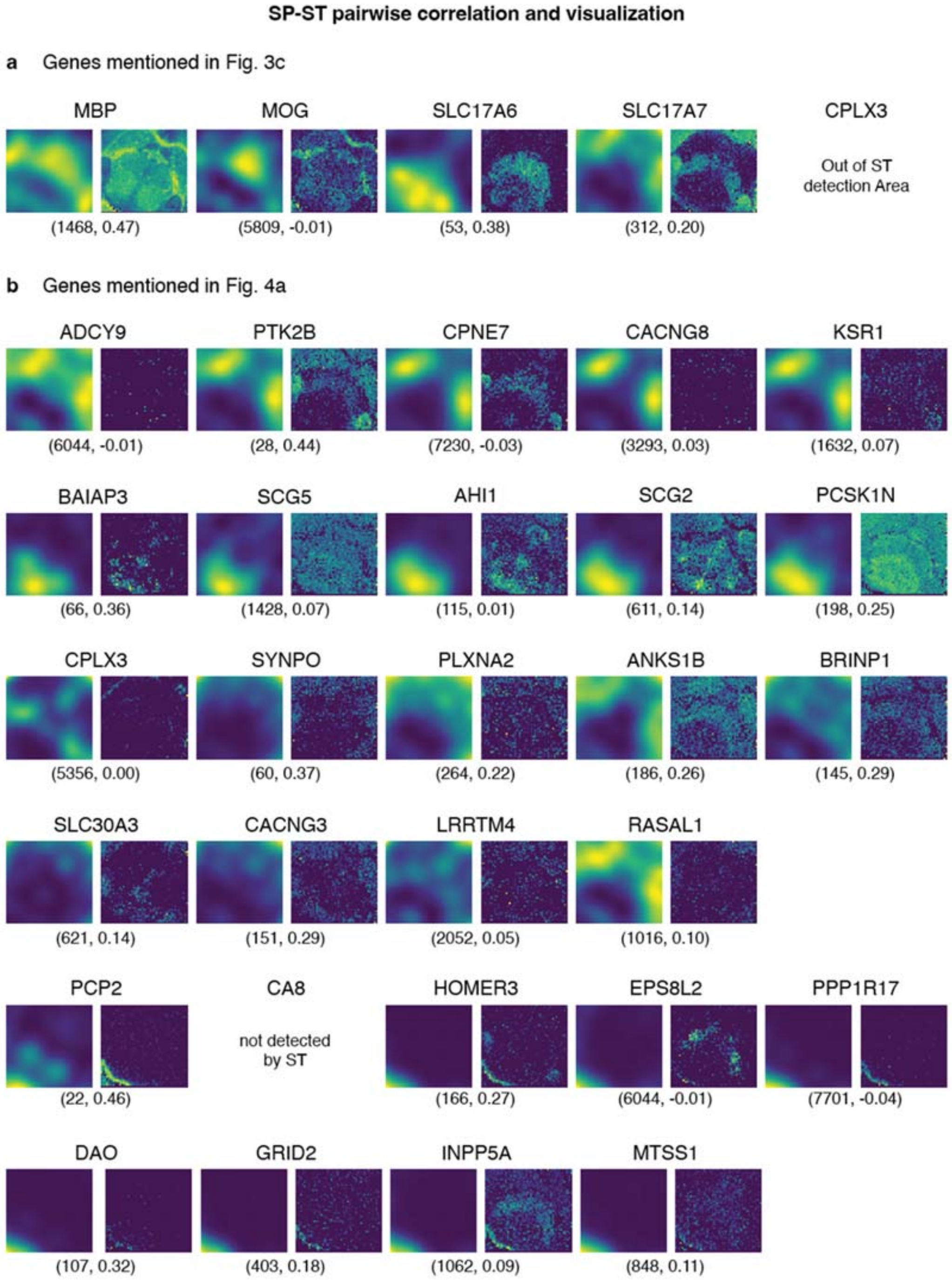

Fig. S14 Pairwise correlation and visualization of SP and ST data.
SP-ST Comparison and visualization of **a** genes mentioned in Figure 3c and **b** Regional markers mentioned in Figure 4a. The rank number of SP-ST correlation in the 8,691 co-identified genes (from the highest correlation to the lowest) is annotated under each SP and ST image. The correlation coefficient is annotated behind the corresponding rank.

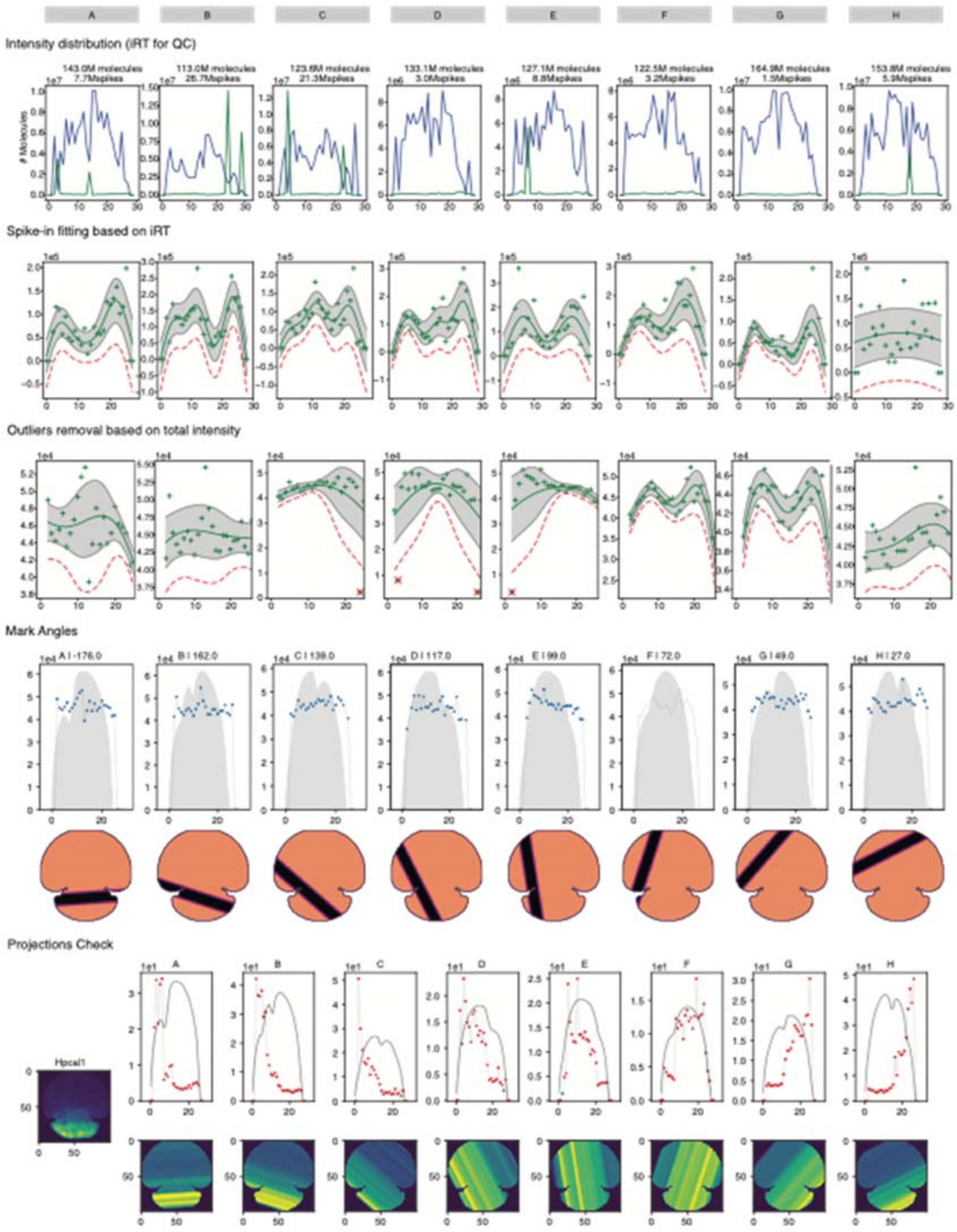

Fig. S15 Data pre-processing before spatial reconstruction.

Total MS intensity of the iRT peptides used as spike-in for QC and low-quality strips removal. Dissecting angles of groups A to H (-176°, 162°, 139°, 117°, 99°, 72°, 49°, and 27°).

Running Date	Protein Groups	Peptides	Precursors
20220831	7,186	70,912	84,580
20220904	7,160	67,816	79,563
20220906	7,215	68,012	81,177
20220908	7,180	68,393	81,374
20220910	7,203	68,027	80,637
20220912	7,176	67,710	78,880
20220914	7,053	65,401	75,745
20220916	7,092	66,514	78,179
20220918	7,093	66,537	77,885

Table S1. Mass spectrometry quality control using tryptic digested HeLa whole cell lysate.

Prior Knowledge	Sampling Approach	Method	Tissue Type	Pixel Size (μm³)	LC-MS (Effective gradient)	Data Acquisition Mode	Software	Peptides	Protein Groups	Ref
Dependent	Biopsy punch	On-tissue hydrogel-based method	Fresh-frozen rat liver	$357^a \times 12$ / $259^a \times 12$	Orbitrap Fusion Tribrid / 120 min	DDA	X! Tandem	NR	708 / 671	[58]
Dependent	LCM	LCM-nanoPOTS	Fresh-frozen human colon tumor	200 × 200 × 12	Orbitrap Fusion Lumos Tribrid / 115 min	DDA	MaxQuant (1.5.3.30)	~ 7,000	1,827	[59]
Dependent	LCM	LCM-nanoPOTS	Fresh-frozen mouse uterine	100 × 100 × 12	QExactive Plus / 75 min	DDA	MaxQuant (1.5.3.30)	> 12,000	> 2,000	[17]
Dependent	LCM	LCM-SISPROT	Fresh-frozen human colon tumor	5,000,000 × 10	Orbitrap Fusion	DDA (5 fractions)	MaxQuant (1.5.5.1)	NR	2,140-5,271	[60]
Dependent	LCM	LCM-SP3	FFPE human brain	1,000,000-3,000,000 × 10	Orbitrap Fusion Tribrid / 210 min	DDA	MaxQuant (1.6.2.3)	53,475	5,677	[61]
Dependent	LCM	LCM-FASP	Fresh-frozen mouse lung	4,000,000 × 16	QExactive Plus / 225 min	DDA	MaxQuant (1.5.2.8)	39,044	3,446	[62]
Dependent	LCM	DVP	FFPE sample of salivary gland and melanoma	80,000-160,000 × 2.5	timsTOF Pro / 55 min	DDA/DIA	MaxQuant (1.6.7.0)/ DIA-NN (1.8)	NR	3,653	[16]
Dependent	Macrodissection (Razor-blade scrap)	TFE-based method	FFPE human ovary tumor	5,000 × 5,000 × 10	QExactive HF-X / 95 min	DIA	Spectronaut (12.0.20491.17)	~ 35,000 to 40,000	~ 5,000	[63]
Dependent	Biopsy punch	ProteomEx	hydrogel-based tissue expansion of mouse brain	$330^a \times 30$	timsTOF Pro / 50*2 min	PulseDIA	FragPipe (15.0) (MSFragger, 3.1.1)	51,203	6,233	[18]
Independent	Micro-scafford	MASP	Fresh-frozen mouse brain	400 × 400 × 1,000	Orbitrap Fusion Lumos Tribrid / 115 min	DDA	UHR-IonStar	NR	5,019	[23]
Independent	LCM	LCM-SP3	Fresh-frozen human brain tumor	833 × 833 × 10	timsTOF Pro / 17 min	DIA	MaxQuant (1.6.14.0)	NR	32-4,741	[22]
**Independent**	**LCM**	**S4P**	**Fresh-frozen mouse brain**	**~525 × 525 × 80**	**timsTOF Pro / 60 min**	**DIA**	**Spectronaut (16.2)**	**234,768**	**9,318**	**Ours**

Table S2. Comparison of peptide and protein identification using different spatial proteomics approaches.
Pixel Size, the volume of each sample; [a]diameter of a round specimen from a tissue sample; NR, not reported.

Name	iRT peptide Sequences	iRT
iRT-pep a	LGGNEQVTR	-24.92
iRT-pep b	GAGSSEPVTGLDAK	0.00
iRT-pep c	VEATFGVDESNAK	12.39
iRT-pep d	YILAGVENSK	19.79
iRT-pep e	TPVISGGPYEYR	28.71
iRT-pep f	TPVITGAPYEYR	33.38
iRT-pep g	DGLDAASYYAPVR	42.26
iRT-pep h	ADVTPADFSEWSK	54.62
iRT-pep i	GTFIIDPGGVIR	70.52
iRT-pep k	GTFIIDPAAVIR	87.23
iRT-pep l	LFLQFGAQGSPFLK	100.00

Table S3. Sequence and theoretical retention time of the iRT peptides

Summary	# of Spots Under Tissue	4,992
	Mean Reads per Spot	62,913
	Median Genes per Spot	3,059
Sequencing	Number of Reads	314,064,185
	Valid Barcodes	97.9%
	Valid UMIs	100%
	Sequencing Saturation	85.5%
	Q30 Bases in Barcode	94.3%
	Q30 Bases in RNA Read	87.6%
	Q30 Bases in UMI	93.5%
	Q30 Bases in Barcode	94.3%
Mapping	Reads Mapped to Genome	93.1%
	Reads Mapped Confidently to Genome	90.3%
	Reads Mapped Confidently to Intergenic Regions	3.9%
	Reads Mapped Confidently to Intronic Regions	1.5%
	Reads Mapped Confidently to Exonic Regions	84.9%
	Reads Mapped Confidently to Transcriptome	82.8%
	Reads Mapped Antisense to Gene	0.7%
Spots	Fraction Reads in Spots Under Tissue	100.0%
	Mean Reads per Spot	62,913
	Mean Reads Under Tissue per Spot	61,603
	Median UMI Counts per Spot	6,865
	Median Genes per Spot	3,059
	Total Genes Detected	20,628
Sample	Slide Serial Number	V11T16-076-D1
	Pipeline Version	spaceranger-1.3.1

Table S4. Summary of the Spatial Transcriptome (ST) Data

Supplementary video titles: Introduction of S4P method
